# A Scoping Review of GLP-1 Receptor Agonists: Are They Associated with Increased Gastric Contents, Regurgitation, and Aspiration Events?

**DOI:** 10.3390/jcm13216336

**Published:** 2024-10-23

**Authors:** Marvin G. Chang, Juan G. Ripoll, Ernesto Lopez, Kumar Krishnan, Edward A. Bittner

**Affiliations:** 1 Department of Anesthesia, Critical Care, and Pain Medicine, Massachusetts General Hospital, Harvard Medical School, Boston, MA 02114, USA; j.ernlopez@icloud.com (E.L.); ebittner@mgb.org (E.A.B.); 2 Department of Anesthesiology and Perioperative Medicine, Mayo Clinic, Rochester, MN 55905, USA; ripollsanz.juan@mayo.edu; 3 Division of Gastroenterology, Massachusetts General Hospital, Harvard Medical School, Boston, MA 02114, USA; kkrishnan@mgh.harvard.edu

**Keywords:** GLP-1 receptor agonists, aspiration, regurgitation, gastric contents, gastric emptying, anesthesia, anesthesiology, guidelines, guidance, surgery, procedures, perioperative management

## Abstract

**Background:** The increased popularity and ubiquitous use of glucagon-like peptide-1 receptor agonists (GLP-1 RAs) for the treatment of diabetes, heart failure, and obesity has led to significant concern for increased risk for perioperative aspiration, given their effects on delayed gastric emptying. This concern is highlighted by many major societies that have published varying guidance on the perioperative management of these medications, given limited data. We conducted a scoping review of the available literature regarding the aspiration risk and aspiration/regurgitant events related to GLP-1 RAs. **Methods:** A librarian-assisted search was performed using five electronic medical databases (PubMed, Embase, and Web of Science Platform Databases, including Web of Science Core Collection, KCI Korean Journal Database, MEDLINE, and Preprint Citation Index) from inception through March 2024 for articles that reported endoscopic, ultrasound, and nasogastric evaluation for increased residual gastric volume retained food contents, as well as incidences of regurgitation and aspiration events. Two reviewers independently screened titles, abstracts, and full text of articles to determine eligibility. Data extraction was performed using customized fields established a priori within a systematic review software system. **Results:** Of the 3712 citations identified, 24 studies met eligibility criteria. Studies included four prospective, six retrospective, five case series, and nine case reports. The GLP-1 RAs reported in the studies included semaglutide, liraglutide, lixisenatide, dulaglutide, tirzepatide, and exenatide. All studies, except one case report, reported patients with confounding factors for retained gastric contents and aspiration, such as a history of diabetes, cirrhosis, hypothyroidism, psychiatric disorders, gastric reflux, Barrett’s esophagus, Parkinson’s disease, dysphagia, obstructive sleep apnea, gastric polyps, prior abdominal surgeries, autoimmune diseases, pain, ASA physical status classification, procedural factors (i.e., thyroid surgery associated with risk for nausea, ketamine associated with nausea and secretions), and/or medications associated with delayed gastric emptying (opioids, anticholinergics, antidepressants, beta-blockers, calcium channel blockers, DPP-IV inhibitors, and antacids). Of the eight studies (three prospective and five retrospective) that evaluated residual contents in both GLP-1 users and non-users, seven studies (*n* = 7/8) reported a significant increase in residual gastric contents in GLP-1 users compared to non-users (19–56% vs. 5–20%). In the three retrospective studies that evaluated for aspiration events, there was no significant difference in aspiration events, with one study reporting aspiration rates of 4.8 cases per 10,000 in GLP-1 RA users compared to 4.6 cases per 10,000 in nonusers and the remaining two studies reporting one aspiration event in the GLP-1 RA user group and none in the non-user group. In one study that evaluated for regurgitation or reflux by esophageal manometry and pH, there was no significant difference in reflux episodes but a reduction in gastric acidity in the GLP-1 RA user group compared to the non-user group. **Conclusions:** There is significant variability in the findings reported in the studies, and most of these studies include confounding factors that may influence the association between GLP-1 RAs and an increased risk of aspiration and related events. While GLP-1 RAs do increase residual gastric contents in line with their mechanism of action, the currently available data do not suggest a significant increase in aspiration and regurgitation events associated with their use and the withholding of GLP-1 RAs to reduce aspiration and regurgitation events, as is currently recommended by many major societal guidelines. Large randomized controlled trials (RCTs) may be helpful in further elucidating the impact of GLP-1 RAs on perioperative aspiration risk.

## 1. Introduction

The increased popularity and ubiquitous use of glucagon-like peptide-1 receptor agonists (GLP-1 RAs) for the treatment of diabetes, heart failure, and obesity has led to significant concern for increased risk for perioperative aspiration and regurgitation events, given their most notable side effect of delayed gastric emptying which increases gastric contents [1,2,3,4,5,6,7,8,9,10]. However, the data on the extent of their impact on gastric contents and clinically significant regurgitation and aspiration events remains conflicting. This concern about regurgitation and aspiration is important because regurgitation can lead to aspiration events, which are associated with significant perioperative morbidity and mortality. These complications include acute respiratory failure, multiple organ failure, prolonged and unexpected hospital stays—often requiring intensive care—and even death [11,12,13]. This has led to speculation about whether these medications, many of which have long half-lives on the orders of a week, should be held for prolonged periods of time prior to procedures requiring anesthesia and the potential need to delay elective procedures to mitigate risks associated with aspiration [9,10]. This concern raised by anesthesiologists and other healthcare providers is highlighted by many major societies that have published varying guidance on the perioperative management of these medications [14,15,16,17,18,19,20,21,22,23,24,25,26,27,28,29,30]. This guidance is variable and mostly based on expert opinion and limited evidence. Current guidance on GLP-1 RAs by various societies ranges from no significant changes required in the perioperative management of these medications to an abundance of caution with a low threshold to delay cases. Such delays may significantly impact many overstretched healthcare systems struggling to provide quality patient care in a timely fashion. Thus, there is a need for a comprehensive review of the literature regarding the aspiration risk and aspiration events related to GLP-1 RAs.

Here, we provide the most comprehensive scoping review of the literature to date regarding GLP-1 RAs, particularly semaglutide, liraglutide, lixisenatide, tirzepatide, and exenatide, and their associated risks for patients undergoing anesthesia. We examine the literature on endoscopic, ultrasound, and nasogastric(NGT)/orogastric(OGT) evaluation for increased gastric contents suggestive of increased aspiration risk and increased regurgitation and aspiration events. We also examine confounding factors that may be associated with these findings observed in the studies of GLP1 agonists. This scoping review summarizes the rapidly expanding and sometimes conflicting literature in this area to inform practitioners to optimize clinical decision-making and improve patient safety.

## 2. Methods

This scoping review was conducted by a team with expertise in anesthesiology, critical care, and systematic review methodology to ensure a comprehensive review of the existing evidence regarding the use of GLP-1 RAs and their effects on delayed gastric emptying and aspiration risk with the aim to inform and guide clinical practice in the management of patients requiring anesthesia. The review adhered to the review methodology outlined by Grant and Booth and the Preferred Reporting Items for Systematic Reviews and Meta-Analyses extension for Scoping Reviews (PRISMA-ScR) checklist [31,32]. Of note, a review protocol was published prior to starting our study. Covidence systematic review software (Melbourne, VIC, Australia; https://www.covidence.org) was accessed on 10 March 2024 to import studies and employed throughout the review process for title and abstract screening, full-text review, and data extraction.

The review focused on the impact of GLP-1 RAs on delayed gastric emptying and the risk of aspiration or delayed gastric emptying in adult patients, particularly as able to be assessed by endoscopic evaluation for retained gastric food contents, ultrasound evaluation for aspiration risk, and nasogastric tube (NGT) suctioning of gastric contents to evaluate for high gastric residuals. The aim was to summarize the available literature up to 10 March 2024, to understand (1) how GLP-1 RA therapy influences the risk of delayed gastric emptying and aspiration as evaluated by using evidence obtained from endoscopic, ultrasound, and/or NGT/OGT procedures suggesting elevated gastric food content and/or high residual gastric contents and (2) the reported aspiration and/or regurgitation events and outcomes of patients receiving GLP-1 RAs.

### 2.1. Search Strategy

A librarian-assisted search was conducted from inception to 10 March 2024, across multiple databases, including PubMed, Embase, and Web of Science Platform Databases, including Web of Science Core Collection, KCI Korean Journal Database, MEDLINE, and Preprint Citation Index. This search aimed to cover all relevant literature available up to that date, utilizing a detailed set of keywords and phrases associated with GLP-1 RAs and their potential impact on gastric emptying and aspiration risk. The search strategy used the following terms: (semaglutide OR ‘GLP-1 receptor agonist’ OR ‘GLP-1 agonist’ OR ‘GLP1 receptor agonist’ OR ‘GLP1 agonist’ OR Ozempic OR Wegovy OR Rybelsus OR Saxenda OR Victoza OR Trulicity OR Byetta OR Bydureon OR Adlyxine OR Tanzeum OR liraglutide OR dulaglutide OR exenatide OR lixisenatide OR albiglutide) AND (‘gastric emptying’ OR ‘gastric residual’ OR residual OR aspirated OR suctioned OR ‘residual gastric’ OR residue OR suctioned OR ‘gastric volume’ OR ‘retained gastric content’ OR ‘cross-sectional area’ OR CSA OR ‘ultrasound’ OR aspiration OR aspirated OR regurgitation OR regurgitated). The strategy was designed to capture the broadest range of clinical research available through 10 March 2024 regarding aspiration risk and aspiration/regurgitant events related to GLP-1 RAs.

### 2.2. Inclusion and Exclusion Criteria

Studies were selected based on the following criteria: (1) investigations into the association between GLP-1 RA therapy and the risk of aspiration or delayed gastric emptying in adult patients, specifically through endoscopic, ultrasound, and/or NGT/OGT evaluation for retained gastric food and/or high residual gastric contents; and (2) reported aspiration events of patients taking GLP-1 RAs undergoing anesthesia. Exclusion criteria included articles not in English and formats where original research was not presented (e.g., reviews, editorials without original research, conference proceedings, and abstracts). Communications and editorials presenting case series and reports were included in this review.

### 2.3. Data Abstraction

Two reviewers (MGC, EAB) independently screened the titles and abstracts of identified articles to determine eligibility. The same two reviewers then performed full-text reviews, with conflicts resolved by an independent third reviewer (EL). Covidence was customized for this project’s specific aims and tested on a subset of references to ensure uniform application of the criteria noted above. Data extracted included details related to the publication, including authors, year of publication, journal of publication, type of study, patient demographics, confounding factors, details of the GLP-1 RA medication regimen, symptoms present at the time of procedural evaluation, methods and findings related to gastric emptying or aspiration risk (endoscopic, ultrasound, and nasogastric evaluation for increased residual gastric volume retained food contents), details of aspiration and regurgitation events, and any notable outcomes.

## 3. Results

**Figure 1:**
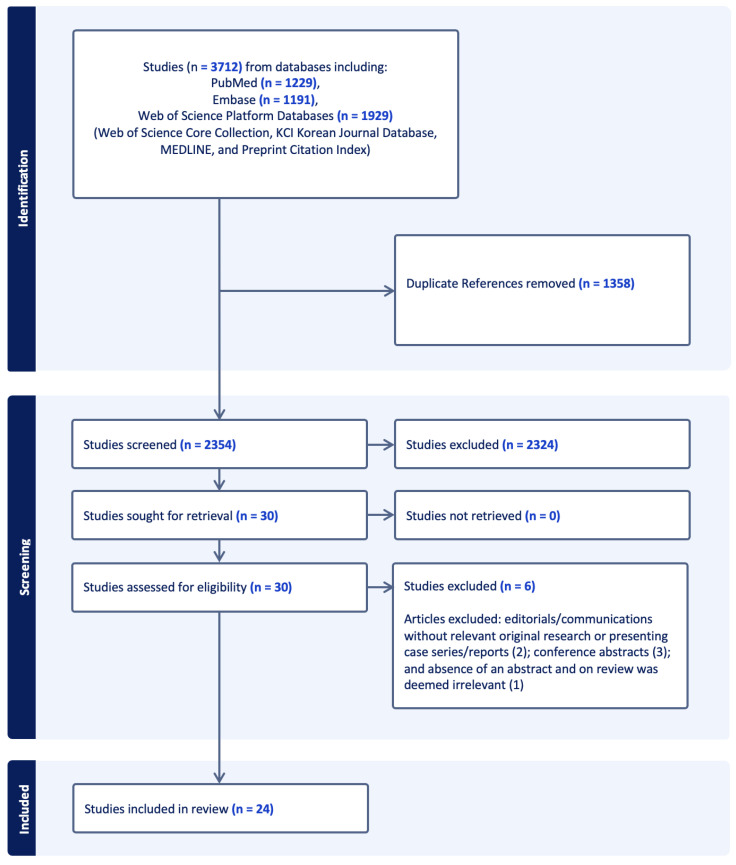
Study extraction and inclusion diagram. EMBASE = Excerpta Medica dataBASE.

The combination of search terms with selection criteria and limits yielded 3712 articles. Of these articles, 1358 duplicates were removed, leaving 2354 articles for title and abstract screening (Figure 1). Of these, 2324 were excluded for not meeting initial inclusion and exclusion criteria, resulting in 30 articles for full-text screening. After the full-text screening, 6 articles were excluded (2 excluded given editorial, 1 one was reviewed due to the absence of an abstract and was deemed irrelevant, and 3 were conference abstracts), resulting in 24 articles being included in the final review, comprising 4 prospective studies [33,34,35,36], 6 retrospective studies [37,38,39,40,41,42], 5 case series [43,44,45,46,47], and 9 case reports [48,49,50,51,52,53,54,55,56].

Table 1 summarizes the characteristics of the 24 articles that met inclusion and exclusion criteria, including author, year of publication, study type, patient demographics, confounding factors, details of the GLP-1 RA medication regimen, symptoms present at the time of procedural evaluation, methods and findings related to gastric emptying or aspiration risk, details of aspiration events, and any notable outcomes [33,34,35,36,37,38,39,40,41,42,43,44,45,46,47,48,49,50,51,52,53,54,55,56]. These studies were published from as early as 2017 to as recently as March 2024, all by separate authors.

We summarize below in detail the type and scale of studies, confounding factors, GLP-1 agonist holding duration, gastrointestinal symptoms, and endoscopic, ultrasound, and nasogastric evaluation for increased residual gastric volume retained food content, as well as aspiration or regurgitant events and outcomes, reported in these studies and also described in Table 1. The Supplementary Text S1 summarizes the detailed information on these studies found in Table 1 related to age and sex demographics, BMI distribution, medication regimens, medication dosing regimens and durations, indication of GLP-1 RA, and fasting time prior to procedures.

### 3.1. Types and Scale of Studies

The article included four prospective studies (one randomized, bicentric, investigator-blinded, parallel group prospective study, two prospective observational studies, and one prospective cross-sectional study) [33,34,35,36], six retrospective studies (one retrospective matched pair case-control study, one retrospective cohort study with matched controls, two retrospective observational studies, and two retrospective cohort studies) [37,38,39,40,41,42], five case series [43,44,45,46,47], and nine case reports [48,49,50,51,52,53,54,55,56].

The size and details of the studies varied significantly across the various types of studies. Of the prospective studies, the cross-sectional study by Sen et al. was the largest prospective study with 124 participants (62 each in GLP-1 RA user and control non-user groups), which compared residual gastric contents measured by gastric ultrasound in GLP-1 RA users and non-user patients presenting for elective surgery, as well as examining the association between duration of interruption of GLP-1 RA and residual gastric contents [35]. Gastric ultrasound was also used to evaluate the effects of GL1-RA on gastric emptying in a study by Sherwin et al., who performed a prospective observational study of 20 volunteer participants (10 on GLP-1 RAs and 10 non-user controls) [36]. Nakatani et al. performed a prospective observational study with 15 patients and investigated the effects of gastric motility using capsule endoscopy prior to and after GLP-1 RA use in patients with diabetes and compared their effects on patients with and without diabetic neuropathy [33]. Quast et al. conducted a randomized, investigator-blinded study with 57 patients involving esophageal manometry studies performed prior to and after starting GLP-1 RAs and reported the incidence of gastroesophageal reflux episodes and pH [34].

Of the retrospective studies, the retrospective, multiple hospital cohort study by Anazco et al. was the largest retrospective study, which assessed the aspiration rate in 2968 unique patients who underwent a total of 4134 endoscopy procedures where GLP-1 RAs were prescribed prior to endoscopies and compared their aspiration rate in GLP-1 RA users to previously published data for non-users [37]. A retrospective study by Bi et al. included a comprehensive cohort of 2150 patients, studying the effects of various medications known to impair gastric emptying (GLP-1 RAs, antacids, cardiovascular medications, and others) on retained gastric food observed on endoscopy [38]. Of note, GLP-1 RAs comprised only a small and unspecified fraction of the patients in the study. A retrospective matched pair case-control study by Kobori et al. involved 1128 individuals, offering an in-depth comparative analysis between the GLP-1 RA and non-user control groups [39]. Additionally, Stark et al. presented findings from a retrospective cohort study with matched controls, comprising 177 individuals (59 patients on GLP-1 RAs and 118 non-user controls), on retained food content and need for gastric lavage or repeat endoscopies due to inadequate visualization on prior endoscopies [41]. Silveira et al. performed a retrospective observational study of 404 patients (33 in the GLP-1 RA group and 371 in the non-user group) that studied gastric residual content volume during endoscopy [40]. Wu et al. performed a retrospective cohort study to assess the residual gastric content on endoscopy of 64 patients on GLP-1 RAs and non-user controls at the time of the endoscopy; the control group patients all started GLP-1 RAs within 1000 days after their endoscopy [42].

The five case series reported were of small size, with one reporting a series of three patients and the other four reporting two patients, including one clinical communication to the editor [43,44,45,46,47]. There were nine case reports included in our review, of which one was a communication to the editor [48,49,50,51,52,53,54,55,56].

### 3.2. Confounding Factors

With the exception of the case report by Weber et al. [56], all of the studies (23 of 24) reported confounding factors for the included patients, which included medical conditions, surgical history, medications, and procedural related influences that may be associated with delayed gastric emptying, aspiration, and regurgitation events. These confounding factors included a history of diabetes, cirrhosis, hypothyroidism, psychiatric disorders, gastric reflux, Barrett’s esophagus, Parkinson’s disease, dysphagia, obstructive sleep apnea, gastric polyps, prior abdominal surgeries, autoimmune diseases, pain, ASA physical status classification, procedural factors (i.e., thyroid surgery associated with risk for nausea, ketamine associated with nausea and secretions), and/or medications associated with delayed gastric emptying (opioids, anticholinergics, antidepressants, beta-blockers, calcium channel blockers, DPP-IV inhibitors, and antacids). While all of the retrospective and prospective studies had exclusion criteria that addressed some, but not all, of the confounding factors mentioned above, each study included patients with at least one of these confounding factors [33,34,35,36,37,38,39,40,41,42]. The case report by Weber et al. contained limited information regarding the patient’s past medical condition; however, it did specifically note that the patient did not have diabetes [56].

Most studies (19 out of 24) considered diabetes a confounding factor. All prospective and retrospective studies included patients with diabetes and did not exclude all diabetic patients from their GLP-1 RA user group, except for the study by Bi et al., which excluded patients with diabetes from their GLP-1 RA analysis group [38]. In the prospective study by Sherwin et al., which evaluated the effects of GLP-1 RAs on gastric emptying using ultrasound, the GLP-1 agonist group included one patient with diabetes, while the control group had no diabetic patients. Notably, patients with Parkinson’s disease, multiple sclerosis, amyloidosis, and scleroderma were not specifically excluded from the study [36]. Moreover, in the study by Quast et al., which used esophageal pH and manometry to evaluate the frequency and severity of reflux episodes in patients before and after GLP-1 RA treatment, all participants took GLP-1 RAs for type 2 diabetes. The exclusion criteria included patients with decompensated diabetes (hemoglobin A1c > 10%) and those with concomitant diseases associated with severe diabetes, such as liver, renal, and hepatic disease [34]. The prospective study by Nakatani et al. included patients with type 2 diabetes, but the exclusion criteria comprised patients with type 1 diabetes, type 2 diabetes on insulin, and marked dysautonomia [33]. All case series, except for two, included patients with a history of type 2 diabetes. The exceptions were the study by Avraham et al., which included only one of two patients with diabetes, and the study by Raven et al., which featured one patient with a history of esophagitis and hypothyroidism, and another patient with a history of gastric reflux and autoimmune disease [43,44,45,46] All of the case reports included patients with diabetes, except for four studies: Weber et al., Gulak et al., Klein et al., and Espinoza et al. [49,50,51,52,54,55,56] The latter three studies involved aspects of the patients’ medical and medication histories that served as confounding factors. Although the study by Weber et al. did not report any confounding factors, it provided very few details about the patient’s medical history, noting only that the patient had recently started the GLP-1 RA for weight loss and did not specifically have diabetes [56].

### 3.3. Holding GLP-1 Agonist Duration

Of the four prospective studies, the GLP-1 RA holding time in the two studies by Quast et al. and Sherwin et al. was not specifically mentioned [34,35,36]. In the study by Naktani et al., the GLP-1 RA was not withheld and was taken on the day of the capsule endoscopy [33]. Lastly, in the study by Sen et al., all but 7 of the 62 patients taking GLP-1 RAs had taken the medication within the last 7 days, while the remaining patients withheld their GLP-1 RAs for 8 to 15 days [35].

Of the six retrospective studies, the GLP-1 RA holding time in the study by Silveira et al. was a mean of 10 days, with patients being instructed to hold the GLP-1 RA for 10–14 days. However, some could not follow instructions for various reasons (i.e., last minute notice to fill case cancelation) [40]. The five remaining studies by Kobari et al., Stark et al., Bi et al., Anazco et al., and Wu et al. did not specifically mention the GLP-1 RA holding time, with Wu et al. specifically noting that no instructions were given to patients to withhold GLP-1 RAs prior to the procedure [37,38,39,41,42].

For the five case series, the duration of holding GLP-1 RA was not mentioned in the study by Weber et al. [56]; one patient had their semaglutide for 6 days, and the other patient held their semaglutide for 4 days prior to the procedure in the study by Abraham et al. [43]. One patient only held the oral semaglutide on the morning of surgery, while the other patient had taken his dulaglutide injection in the past week in the study by Wilson et al. [47]. The GLP-1 RA was not held in all three patients taking weekly subcutaneously semaglutide in the study by Kittner et al. [45], and the GLP-1 RA was taken the day before surgery in one patient where solid food was found on initial esophagogastoduodenoscopy (EGD) and then withheld his GLP-1 RA for 3 weeks prior to repeat EGD, which revealed empty stomach, with the other patient with unspecified GLP-1 RA holding time in the study by Raven et al. [46].

Of the nine case reports, the duration of holding the GLP-1 agonist was not mentioned in the five studies by Almustanyir et al., Rai et al., Ishihara et al., Klein et al., and Weber et al. [48,53,54,55,56]. In contrast, four studies by Fujino et al., Gulak et al., Espinoza et al., and Giron-Arango et al. [49,50,51,52] reported that the medication was not withheld and was taken as prescribed. Notably, in the study by Fujino et al., the patient underwent a repeat endoscopy after the initial upper EGD was aborted due to the presence of food residue in the stomach. For the repeat endoscopy, the patient took semaglutide seven days earlier and was instructed to follow a liquid diet for 36 h before the procedure [50].

### 3.4. GI Symptoms on Presentation

Of the four prospective studies, the gastrointestinal symptoms in the study by Quast et al. were reported to include heartburn, nausea, vomiting, diarrhea, or loose stools in both patient groups treated with lixisenatide and liraglutide, and no differences between the two groups [34]. The three studies by Sherwin et al., Nakatani et al., and Sen et al. did not evaluate or mention symptoms on the day of the procedure, with the Nakatani et al. study reporting that digestive symptoms induced by liraglutide such as nausea and diarrhea were not severe and did not require discontinuation of the drug in any patients [33,34,35,36]. In the six retrospective studies, gastrointestinal symptoms in the study by Silveira et al. were present in 27.3% of patients in the GLP-1 RA group and 4.5% in the non-user group [40]. In the study by Wu et al., they were present in 12% of patients in the GLP-1 RA group and 2% in the non-user group [42], and they were not specifically mentioned in the four studies by Kobori et al., Stark et al., Bi et al., and Anazco et al., where the indication for endoscopies was not specifically mentioned except in the Stark et al. study, which specifically mentioned that the indications included anemia, gastric reflux, Barrett’s esophagus, dysphagia, abdominal pain, history of gastric polyps, surgical screening, and other [37,38,39,41]. Of the five case series, gastrointestinal symptoms in the two studies by Kittner et al. and Kalas et al. were present and included nausea, vomiting, abdominal bloating, fullness, and/or postprandial epigastric pain [44,45]. The study by Wilson et al. had both patients without gastrointestinal symptoms [47]. The study by Avraham et al. specifically mentioned no nausea and vomiting in one patient and did not specifically mention gastrointestinal symptoms in the other patient [43], and in the study by Raven et al., it was not specifically mentioned. However, one patient’s endoscopy was routine follow-up for esophagitis and the second patient’s indication for endoscopy was reflux [46]. Of the nine case reports, gastrointestinal symptoms—such as nausea, vomiting, abdominal distention, decreased appetite, and weight loss—were present in three studies [48,53,55]. In two studies, these symptoms were specifically reported as absent [50,51,52], and in four studies, gastrointestinal symptoms were not specifically mentioned [49,50,51,54,55,56].

### 3.5. Endoscopic, Ultrasound, and Gastric Volume Findings

Of the four prospective studies, capsule endoscopy was used to evaluate gastric time in patients prior to and after GLP-1 RAs in one study by Nakatani et al. [33]. Gastric ultrasound was used to assess the effects of gastric emptying in patients taking GLP-1 RAs and control non-users in two studies by Sherwin et al. and Sen et al. [35,36]. There was no evaluation for retained gastric contents or increased gastric residual content by endoscopic, ultrasound and/or nasogastric or orogastric evaluation in the study by Quast et al. [34]. In the study by Nakatani et al., gastrointestinal residue rates were significantly increased post-liraglutide compared prior to starting liraglutide in both the diabetic neuropathy (90.0 ± 9.1% vs. 32.1 ± 24%, *p* < 0.001) and non-diabetic neuropathy (78.3 ± 23.9% vs. 32.1 ± 35.3%, *p* < 0.001) groups, while gastric, duodenal, and small intestine transit times were significantly increased after liraglutide administration compared to before starting liraglutide in patients with non-diabetic neuropathy but not in patients with diabetic neuropathy [33]. In the Sherwin et al. study, which involved assessing for solid gastric contents using gastric ultrasound, 70% and 90% of GLP-1 RA participants compared to 10 and 20% of non-user participants in the supine and lateral positions, respectively, showed solid gastric contents after an 8 h fast; participants subsequently had a gastric ultrasound study performed two hours after clear liquid intake which revealed no difference in the lateral position but a significant difference in the supine position, where 90% of controls had an empty stomach compared to 30% of the GLP-1 RA group [36]. On gastric ultrasound imaging, the semaglutide group’s gastric solids were noted to have a layered/yogurt-like consistency. In the study by Sen et al., retained gastric contents evaluated by preprocedural gastric ultrasound were higher in the GLP-1 RA group compared to the non-user group (56% versus 19%), and there was no significant association between the duration of withholding GLP-1 RA and prevalence of retained gastric contents [35]. The study by Quast et al., which did not evaluate for retained gastric contents or increased gastric residual content by endoscopic, ultrasound, and/or nasogastric or orogastric evaluation, involved esophageal pH and manometry studies that evaluated for reflux episode frequency, which is detailed in the following section [34].

Of the six retrospective studies, two found that the occurrence of gastric residue was significantly higher in GLP-1 RA users compared to non-users. Specifically, Kobori et al. reported a prevalence of 5.4% in users versus 0.49% in non-users (*p* = 0.004), while Wu et al. reported 19% in users compared to 5% in non-users (*p* = 0.004) [39,40,41,42]. In the study by Silveira et al., the residual gastric content (defined in their study as any amount of solid content, or >0.8 mL/Kg measured from aspiration/suction canister) was higher in GLP-1 RAs users compared to non-users (24.2% vs. 5.1%, *p* < 0.001) but not the subjective amount of residual gastric contents found on EGD by visual estimation (small, medium, large) [40]. Moreover, the retained gastric content was not statistically significantly higher between the GLP-1 RA users and non-users in the two studies by Stark et al. and Bi et al. Of note, in the latter study, the retained gastric content was found only to be significant on univariate but not multivariate analysis [38,39,40,41]. In the study by Anazco et al., retained gastric contents were not rigorously assessed in the GLP-1 RA user group except in the context of patients with definite pulmonary aspiration events [37]. Notably, in the study by Kobori et al., which found a higher occurrence of gastric residue in GLP-1 RA users compared to non-users, patients with gastric residue were significantly younger and were taking a weekly dose of semaglutide at 1.0 mg. No gastric residue was reported in patients treated with lower doses of liraglutide (≤1.5 mg), lower doses of semaglutide (<0.25 mg), or any dose of oral semaglutide, lixisenatide, or exenatide [39]. In the Wu et al. study, the GLP-1 RA group had 4 of 71 procedures starting as monitored anesthesia care (MAC) with documented residual gastric contents resulting in emergent endotracheal intubation. One of these cases involved a pulmonary aspiration event that led to a transfer to the ICU; the patient was extubated four hours later and discharged the next day. In contrast, no such events were reported in the non-user group [42]. The Stark et al. study, which found that retained gastric content was not statistically different between GLP-1 RA users and non-users, also found that there was no difference in the necessity to perform gastric lavage and undergo a repeat EGD due to inadequate visualization in the GLP-1 RA users compared to non-users [41]. In the Silveira et al. study, propensity weighted analysis revealed increased risk for residual gastric contents with factors such as the use of semaglutides (Prevalence Ratio (PR) = 5.15, 95% CI (1.92–12.92), *p* < 0.001), preoperative digestive symptoms (including nausea/vomiting, dyspepsia, abdominal distension) (PR = 3.56. 95% CI 2.25–5.78, *p* < 0.001), semaglutide use and digestive symptoms (PR = 16.5, 95% CI 9.08–34.91, *p* < 0.001), semaglutide use and no digestive symptoms (PR = 9.68, 95% CI 5.6–17.66, *p* < 0.001), and no semaglutide use and digestive symptoms (PR = 4.94, 95% CI 1.32–15.77, *p* = 0.0098), while patients undergoing both EGD and colonoscopy showed a protective effect against residual gastric contents (PR = 0.25, 95% CI 0.16–0.39, *p* < 0.001) [40]. In the same study by Silveira et al., while the pre-endoscopy digestive symptoms were associated with increased retained gastric contents, the duration of preoperative semaglutide cessation did not impact the presence or absence of retained gastric content [40]. In the study by Anazco et al., which did not rigorously assess gastric residues except in the context of two confirmed pulmonary aspiration events among the 4134 reviewed endoscopic procedures following GLP-1 RA prescriptions, one of the patients with an aspiration event was found to have retained food content in the stomach and duodenum during an upper endoscopy performed under monitored anesthesia care (MAC). The other patient who experienced an aspiration event was noted to have ectasia with bleeding and subsequently underwent argon plasma coagulation (APC) [37].

Of the five case series studies, endoscopy revealed solid food in two patients in the study by Raven et al., with one of the patients in the same study having a repeat endoscopy that revealed an empty stomach after holding their GLP-1 RA for 3 weeks and fasting for 12 h [46]. An endoscopy revealed no obstruction in two patients presenting with gastrointestinal side effects consistent with gastroparesis in the study by Kalas et al. [44]. Gastric ultrasound revealed all three patients with residual solid food for patients presenting for orthopedic procedures (also mentioned data from same institution where seven out of eight patients presenting for non-orthopedic procedures had retained solid food on ultrasound evaluation) in the study by Kittner et al. [45], and there was no mention of endoscopic, ultrasound, or gastric findings in the two studies by Wilson et al. and Avraham et al., with the latter study by Avraham et al. reporting nasogastric suctioning following regurgitant epsidoes in two patients without reporting the actual gastric volume suctioned [43,44,45,46,47]. Of note, in the Kalas et al. study, both patients reported gastrointestinal side effects consistent with gastroparesis and had symptom resolution and normalization of the gastric emptying scintigraphy (GES) studies after their GLP-RA agonist was held for 4–6 weeks [44].

Of the nine case reports, the five of the case reports by Almustanyir et al., Rai et al., Fujino et al., Ishihara et al., and Klein et al. involved endoscopies, of which two of the studies, by Rai et al. and Ishihara et al., had NGTs placed prior to the procedures with reported residuals [48,49,50,53,54,55]. Two of the case reports by Gulak et al. and Weber et al. reported gastric residuals measured by orogastric tube placed following an aspiration and/or regurgitant episode under anesthesia [52,53,54,55,56]. The case report by Giron-Arango et al. reported increased gastric volumes assessed by gastric ultrasound consistent with full stomach [51], and the case report by Espinoza et al. did not assess gastric residuals using ultrasound or nasogastric tube in this patient presenting for ECT [49]. Of the five studies that reported endoscopic findings, three studies, by Alustanyir et al., Rai et al., and Ishihara et al., reported no obstructing lesion [48,53,55], and the two studies by Fujino et al. and Klein et al. revealed food residue in the stomach, with the study by Fujino et al. leading to abortion of EGD and a repeat EGD a month later with no food residue after a prolonged 36 h liquid fast and patient having taken their semaglutide 7 days prior [50,51,52,53,54]. Of the two studies by Rai et al. and Ishihara et al., which had NGTs placed prior to the procedures, the reported suctioned gastric residuals were 1 L and 600 mL, respectively [53,54,55]. Of the two studies which reported gastric residuals following an orogastric tube placed after an aspiration and/or regurgitant episode under anesthesia, the reported residual amounts were minimal in the study by Gulak et al. and increased (750 mL) in the study by Weber et al. [52,53,54,55,56]. Of the three studies by Almustanyir et al., Rai et al., and Ishihara et al., which reported gastrointestinal symptoms, all four patients had resolution of their symptoms with conservative management, which included discontinuation of the GLP-1 RA, NGT placement, antiemetics, and/or prokinetic medications [48,49,50,51,52,53,55].

### 3.6. Aspiration or Regurgitant Events and Outcomes

Of the four prospective studies, the study by Quast et al. revealed that regurgitant events as evaluated by reflux episode frequency and severity, as well as esophageal motility and lower esophageal sphincter functionality using esophageal pH and manometry, were not significantly different between GLP-1 agonist users and non-users despite gastric emptying as evaluated by octanoate acid breath test being more significantly prolonged in the GLP-1 agonist user group compared to the non-user group [34]. In the other three studies, by Nakatani et al., Sherwin et al., and Sen et al., aspiration or regurgitant events were not applicable given that the primary interventions or evaluations were capsule endoscopy (Nakatani et al.) or gastric ultrasound (Sherwin et al. and Sen et al.), and the patients did not receive anesthesia [33,35,36].

Of the six retrospective studies, the three studies by Kobori et al., Stark et al., and Bi et al. did not specifically mention aspiration or regurgitation events [38,39,41]. One study by Anazco et al. reported an aspiration rate of 4.8 cases per 10,000 endoscopies in patients after GLP-1 prescription compared to 4.6 cases per 10,000 endoscopies reported previously in patients presenting for elective endoscopies from 2000 to 2016 using the same previously validated automatic search algorithm of the electronic medical record system by Bohman et al. [37,38,39,40,41,42,43,44,45,46,47,48,49,50,51,52,53,54,55,56,57]. One study by Wu et al. reported one case of pulmonary aspiration in the GLP-1 RA group (total of 90 procedures from 64 patients) and none in the control group (total of 102 procedures from 69 patients) consisting of patients undergoing endoscopies [42], and the study by Silveira et al. reported one case (0.24%) of pulmonary aspiration under deep sedation in patients undergoing elective endoscopies [40]. For the studies by Anazco et al., Wu et al., and Silveira et al., which reported aspiration events, the patient characteristics and outcomes following the aspiration event were reported as follows [37,40,42]. In the study by Anazco et al., the two aspiration events occurred in one patient who was a 47-year-old woman, ASA III, with normal weight, on dulaglutide (3.0 mg weekly, started 30 months ago) for diabetes (HbA1c 6.7) and who had an upper endoscopy for abdominal pain and diarrhea to rule out celiac disease and was found to have retained food contents in the stomach and duodenum during upper endoscopy, had massive vomiting upon scope removal with direct visualization of aspirated contents in the airways and chest imaging compatible with aspiration, and required intubation, ICU admission for 3 days, and vasopressors and was discharged from the hospital after 5 days with oxygen. The other patient was a 72-year-old woman, ASA III, with obesity (class II) and GERD, taking semaglutide (0.5 mg weekly, started 3 months ago) for obesity and had no symptoms on initiation of GLP-1 RA. She had presented for upper endoscopy for iron deficiency anemia and possible GAVE and was found to have diffuse gastric antral vascular ectasia and bleeding requiring APC; she did not have visualization of retained food contents in stomach and duodenum, had persistent hypoxemia after the procedure with imaging compatible with aspiration, did not require ICU admission, and was discharged home after 8 days of hospitalization [37]. In the study by Wu et al., the past medical history and comorbidities of the one patient in the GLP-1 RA group who aspirated was a 42-year-old man with obesity (BMI 37), prior history of Barrett’s esophagus on PPI and histamine receptor agonist, OSA on CPAP, mixed anxiety and depressive disorder on medications with anticholinergic effects (paroxetine), and prior history of lung abscess likely secondary to aspiration in the setting of heavy alcohol use (now sober for 4 years), who had fasted for more than 18 h, with the last dose of GLP-1 RA and gastrointestinal symptoms not reported. He had a pulmonary aspiration event which resulted in emergent intubation and bronchoscopy, revealing food remains in trachea and bronchi; he was transferred to the ICU, extubated 4 h later, and discharged the following day [42]. In the study by Silveira et al., the one reported patient who aspirated in the GLP-1 RA group was a 63-year-old man with obesity (BMI 37.7), prior history of gastric bypass, and semaglutide use (last taken 11 days prior), who had adequately fasted (12.4 h for solids and clear fluids) and reported no digestive symptoms prior to his endoscopy and was not specifically reported to have any negative sequelae following his aspiration event [40].

Of the five case series, two studies by Wilson et al. and Avraham et al. both described two patients who had regurgitant or aspiration events in the perioperative period [43]. One study by Kittner et al. reported postponing the cases of three patients presenting for orthopedic surgery after gastric ultrasound revealed solid food residue [45]. Two studies by Raven et al. and Kalas et al. did not specifically mention an aspiration or regurgitant event during upper endoscopies which revealed residual gastric food content in the Raven et al. study and an absence of obstruction and no specific mention of residual gastric content in the study by Kalas et al. [44,45,46]. In the study by Wilson et al., which described two patients who had regurgitant or aspiration events in the perioperative period, one patient developed orpharyngeal secretions and labored respirations after regional anesthesia and monitored anesthesia care for foot arthrodesis with fentanyl, midazolam, propofol, and ketamine and, following intubation, had significant bilious particulate matter suctioned from the oropharynx and was discharged after 2 h in recovery; the other patient had projectile vomiting with bile-tinted particulate matter following extubation after a thyroidectomy and was discharged the following day per protocol for thyroidectomy procedures [47]. In the study by Avraham et al., one patient had a large volume of regurgitant contents with particular food noted during laryngoscopy following RSI for ERCP, with bilateral infiltrates noted on CXR, and required transfer to ICU, was extubated the following day, and was discharged from the hospital after a week; the other patient had regurgitation of solids and liquids following LMA removal after completion of surgery for I&D of breast abscess, with head tilted and suctioned, rapid sequence intubation, and then extubation awake, and was then transferred to the PACU, with reportedly normal CXR and observation that was normal [43].

Of the nine case reports, an aspiration or regurgitant event was not specifically mentioned in the five studies by Giron-Arango et al., Amustanyir et al., Rai et al., Ishihara et al., and Kobori et al. [39,48,51,53,55], and was noted to not have occurred in two studies, one by Fujino et al. after food residue was found in the stomach during upper endoscopy and the procedure was aborted, and another study by Espinoza et al., where ECT was performed after a risk and benefit discussion with a patient who had last taken his semaglutide days prior [49,50]. Regurgitation or aspiration was noted to have occurred in the three studies by Gulak et al., Klein et al., and Weber et al., where the patients underwent procedures under anesthesia [52,54,56]. The patient in the Gulak et al. case report regurgitated 200 mL of clear fluid after 30 s of gentle mask ventilation following induction of anesthesia; a fiberoptic bronchoscopy revealed no evidence of aspiration and an orogastric tube placed prior to extubation had minimal output [52]. Klein et al. reported a large volume solid and liquid aspiration event during an upper endoscopy; fiberoptic bronchoscopy revealed food remains in the trachea and bronchi. Of note, this was the only aspiration event that was noted in the retrospective study by Wu et al. [42,43,44,45,46,47,48,49,50,51,52,53,54]. In the study by Weber et al., a large volume of gastric aspirate was noted in the oropharynx after supraglottic airway placement for hysterscopy procedure, necessitating tracheal intubation, and orogastric tube placed afterwards revealed approximately 750 cc undigested food and gastric contents [56]. All of the patients reported by Gulak et al., Klein et al., and Weber et al. who had large volume regurgitant or aspiration events were discharged home the following day (of note, they were 42M, 48F, and 59F), with the patient in the Klein et al. study requiring ICU admission [52,54,56].

## 4. Discussion

We present the first comprehensive scoping review of the impact of GLP-1 RA on the incidence of retained gastric food contents or high gastric residuals assessed by endoscopic, ultrasound, nasogastric and/or orogastric evaluation, as well as reported regurgitation and aspiration events. This review includes findings from a diverse array of studies, including four prospective studies [33,34,35,36], six retrospective studies [37,38,39,40,41,42], five case series [43,44,45,46,47], and nine case reports [48,49,50,51,52,53,54,55,56], to synthesize the best available evidence and clinical implications of GLP-1 RA use in patients requiring anesthesia. The majority of the studies were case reports and case series with small sample sizes and low-quality evidence, and many of the studies had significant uncontrolled confounding factors that made it difficult to draw definitive conclusions. From the higher quality of evidence provided by prospective and retrospective studies included in this review, there appears to be insufficient evidence that GLP-1 RAs are consistently associated with an increased incidence of retained gastric contents or regurgitation and aspiration events in the patient populations studied.

It is interesting to note that GLP-1 RAs have been FDA-approved since 2005 for diabetes, and it is only recently when use has become ubiquitous that concern for aspiration has also become widespread [9,10,11,12,13,14,15,16,17,18,19,20,21,22,23,24,25,26,27,28,29,30,31,32,33,34,35,36,37,38,39,40,41,42,43,44,45,46,47,48,49,50,51,52,53,54,55,56,57,58]. Of note, concern shared by the community regarding GLP-1 RAs has not been the same for many other commonly administered medications associated with decreased gastric emptying, including opioids, cardiovascular medications (beta and calcium channel blockers), anticholinergics, antacids, and neurological and psychiatric medications.

### 4.1. Variable Association of GLP-1 RAs with Retained Gastric Contents

There were conflicting reports of retained gastric contents in the higher-quality-of-evidence retrospective and prospective studies reviewed. For the retrospective studies, five of six studies investigated an association of GLP-1 RAs with retained gastric contents. Of these five studies, three studies (Wu et al., Kobori et al., and Silveira et al. [39,40,42]) showed a positive association while the remainder did not (two studies by Stark et al. and Bi et al. [38,39,40,41]). For the prospective studies, only three of the four studies investigated retained gastric contents. All three of these studies showed significant increase in gastric residue rates on either capsule endoscopy after starting GLP-RAs (in the case of the study by Nakatani et al. [33]) or on gastric ultrasound in the GLP-1 user group compared to non-user groups (in the cases of the studies by Sherwin et al. and Sen et al. [35,36]); of note, all three studies reported significant residue rates in the non-GLP-1 RA user groups as high as 32.1 ± 24% and 32.1 ± 35.3% in the diabetic neuropathy and non-diabetic neuropathy groups, respectively, reported in the study by Nakatani et al. [33]. Furthermore, retained gastric contents were found in up to 20% of the non-GLP-1 RA user group assessed by ultrasound in the study by Sherwin et al. and in 19% of the non-GLP-1 RA user group assessed by gastric ultrasound in the Sen et al. study [35,36]. Thus, the high gastric residue rates found in the groups of patients not taking GLP-1 RAs must be considered as a significant confounding factor in the noncontrolled case reports and series reporting increased gastric food contents and increased gastric residuals; the medical conditions and medications associated with delayed gastric emptying in many of these studies also serve as significant confounding factors. It is also important to note that only the study by Bi et al. excluded patients with diabetes in their analysis of GLP-1 RA and retained gastric contents [38]. This study did not identify a statistically significant association between GLP-1 and retained gastric food during upper endoscopy in their multivariate analysis, while opioids, antacids, and cardiovascular medications were associated with retained gastric contents.

### 4.2. Variable Association of GLP-1 RAs with Aspiration and Regurgitant Events

The literature reflects significant variability regarding the association of GLP-1 RAs with regurgitant events and aspiration on review of the higher-quality retrospective and prospective studies. For the retrospective studies, three of the six studies assessed aspiration events. Among these, the study by Anazco et al. revealed a similar aspiration rate of 4.8 cases per 10,000 endoscopies in patients taking GLP-1 RA compared with the 4.6 cases per 10,000 endoscopies previously reported in a prior Mayo Clinic study [37]. In this study, Anazco et al. used a previously validated automatic search algorithm of the electronic medical record system and reported two aspiration events in patients after receiving GLP-1 RA prescription, both of whom had significant confounding factors for aspiration including diabetes (HbA1c 6.7%) in one patient and gastric reflux with diffuse gastric antral vascular ectasia and bleeding requiring APC in the other patient [37]. The remaining retrospective reviews by Wu et al. and Silveira et al. each had a report of a single aspiration event during endoscopy, with the patients who aspirated having significant risk factors for aspiration [40,41,42]. All the patients on GLP-1 RA who aspirated in the retrospective studies survived, with two of the four patients requiring ICU admission (1 and 3 days total), one patient requiring non-ICU hospitalization and one patient being discharged on oxygen.

Only one (Quasi et al. [34]) of the four prospective studies studied regurgitant events evaluated by reflux episode frequency using esophageal pH and manometry. In this study, there was no significant difference for reflux episode frequency between GLP-1 RA users and non-users despite a significant prolonged gastric emptying time on the octanoate acid breath test, suggesting that their risk for reflux or regurgitation associated aspiration events may not be increased with GLP-1 RA use [34]. This is important because reflux is a significant risk factor for aspiration and regurgitant events and an important factor anesthesia providers use to determine the need for rapid sequence induction; however, it is important to note that a regurgitant event does not necessarily lead to a significant aspiration event [11]. It is also important to note that the case reports and case series reporting aspiration and/or regurgitant events reported patients that had confounding medical, surgical, anesthesia-related, procedural, and medication-related factors associated with delayed gastric emptying, increased secretions, and post-operative nausea and vomiting. None of the patients in the case reports and series reported any long-term negative sequelae of the aspiration or regurgitant events. All patients that suffered aspiration or regurgitant events were discharged by the following day with the exception of one patient (in the case series by Avraham et al. [43]), who was discharged from the hospital after a week.

Interestingly, we queried the clinicaltrial.gov database for all completed and terminated studies that involved GLP-1 RAs reported in our study (liraglutide, lixisenatide, semaglutide, dulaglutide, tirzepatide, and exenatide) and had reported study results, which revealed a total of 399 studies (372 completed and 27 terminated), of which 12 of the completed studies and none of the terminated studies specifically mentioned aspiration, aspiration pneumonitis, aspiration pneumonia, and foreign body aspiration as an adverse event in their study results; of these 12 studies, 9 of the studies had a control drug or placebo group to compare with the GLP-1 RA group’s incidence of reported adverse events. Our query revealed one study with equal incidence of reported aspiration pneumonia in both the GLP-1 RA and placebo groups (clinicaltrials.gov ID NCT01147250); six studies had higher rates of aspiration and/or aspiration pneumonia in GLP-1 RA group compared to placebo/control (clinicaltrials.gov IDs NCT04184622, NCT03574597, NCT01720446, NCT01144338, NCT02692716, and NCT01621178); and two studies had higher incidences of reported aspiration and/or foreign body aspiration in the placebo group compared to the GLP-1 RA group. (clinicaltrails.gov IDs NCT01179048 and NCT01394952). We also queried the FDA Adverse Events Reporting System (FAERS), which revealed that the GLP-1 RAs reported in our study (liraglutide, lixisenatide, semaglutide, dulaglutide, tirzepatide, and exenatide) reported aspiration, aspiration pneumonitis, and aspiration pneumonia in a total of 146 cases out of 252,418 total adverse event cases reported (0.058%), which was less than that for other medication typically continued in the perioperative period, particularly aspirin, simvastatin, atorvastatin, metoprolol, and carvedilol, which reported aspiration, aspiration pneumonitis, and aspiration pneumonia in a total of 205 cases out of 258,429 total adverse event cases reported (0.079%). It is important to note that the reported data from FAERS does not reflect actual incidence rates, and may be subject to underreporting and reporting bias. Nevertheless, the low number of aspiration-related cases reported in both groups and the results from our scoping review suggests that the risk may be lower than initially perceived and possibly overstated. Further studies are necessary to determine the risk for aspiration and/or regurgitant events relative to other medications and medical/surgical conditions associated with delayed gastric emptying.

### 4.3. Potential Impact of Diabetes and the Severity of Disease

From the larger prospective studies by Nakatani et al., Sherwin et al., and Sen al., it is evident that the gastric residual contents are relatively high even in the non-GLP-1 receptor agonist analysis groups [33,35,36]. These studies did not exclude diabetics in the user and non-user groups, which is informative because the study populations are representative of the real world patient populations often using GLP-1 receptor agonists and undergoing anesthetic procedures. Only the retrospective study by Bi et al. excluded diabetics as part of their GLP-1 receptor agonist and gastric residue analysis, finding no significant association with GLP-1 receptor agonists with retained gastric food during upper endoscopy on multivariate analysis [38]. However, the small number of patients taking GLP-1 receptor agonists in the study may have been a limiting factor in the ability to detect a difference. In the same study by Bi et al., the authors reported that opioids, antacids, and cardiovascular medications were associated with retained gastric food [38]. It is noteworthy to compare the findings from these studies with the Australian and New Zealand College of Anaesthetists (ANZA) guidance indicating that discontinuing GLP-1 receptor agonists does not alter the risk of delayed gastric emptying that is inherently associated with diabetes [16,17,18,19,20,21,22,23,24,25,26].

It is possible that diabetes severity may play a role in the differential response to GLP-1 RA intake and overall risk profile. The prospective study by Nakatani et al. found that, using capsule endoscopy, the gastric, duodenal, and small intestine transit times were significantly increased after liraglutide administration compared to before starting liraglutide in diabetic patients with non-diabetic neuropathy but not in patients with diabetic neuropathy despite showing a significantly increased gastric residue in both nondiabetic neuropathy and diabetic neuropathy diabetic patients [33]. This may reflect increased gastrointestinal side effects in patients without severe diabetes who have not developed gastrointestinal autonomic neuropathy and are taking GLP-1. It is unclear whether this leads to a clinically significant increase in gastric residuals that may place the patient at risk for aspiration. In the Silveira et al. study, the authors found that the perioperative use of GLP-1 RAs was associated with increased residual gastric content, which they defined as any amount of solid content or >0.8 mL/Kg measured from aspiration or suction canister compared to non-users (24.2% vs. 5.1%, *p* < 0.001), but not the subjective amount of residual gastric content found on EGD by visual estimation (small, medium, large) [40]. For a 70 kg person, this threshold for residual gastric content would equate to just 56 mL measured from aspiration or suctioned from the canister. This is well below the 500 mL threshold used in critical care settings, where gastric residuals above this amount raise concerns for ileus and often result in the discontinuation of tube feeds [59]. While many of the case reports did report high gastric residuals of greater than 500 mL when assessed with nasogastric or orogastric tube, many of the patients had diabetes and/or did not specifically mention the presence or absence of the patient’s other medical history that may serve as potential confounding factor. The study by Gulak et al. was the only case report or case series where the patient was noted not to be diabetic, specifically mentioned the patient’s other medical issues, and reported regurgitation or gastric residual volumes [52]. In this report, the patient exhibited regurgitation of 200 mL of clear fluid during induction of anesthesia, with minimal gastric content observed after orogastric tube placement. Additionally, the patient has a history of hypothyroidism, which may contribute as a confounding factor for high residual volumes. The remaining case reports and series by Rai et al., Ishihara et al., and Weber et al. evaluated gastric residuals through orogastric or nasogastric tube placement. Both Rai et al. and Ishihara et al. included patients with diabetes, while the patient in the correspondence from Weber et al. did not have diabetes. However, Weber et al. did not provide specific details about the patient’s other medical history, which could also have acted as confounding factors [53,55,56]. Higher-quality retrospective and prospective studies are needed to clarify the risk of clinically significant increases in gastric residuals associated with GLP-1 RAs.

Importantly, withholding GLP-1 RAs in the perioperative period may lead to significant elevations in blood glucose, particularly in patients using GLP-1 RAs for hyperglycemia management. Elevated blood glucose levels are associated with increased perioperative morbidity and mortality and include complications such as impaired wound healing and increased surgical site infection [60,61,62]. Thus, careful consideration of both glycemic control and aspiration risk is critical, and future guidelines should consider balancing these competing risks, providing evidence-based recommendations that prioritize patient safety and optimal surgical outcomes.

### 4.4. Gastrointestinal Symptoms and Risk for Retained Gastric Contents

The impact of gastrointestinal symptoms on retained gastric contents was evaluated in one retrospective study, four case series, and five case reports. In the retrospective study of patients undergoing upper endoscopies by Silveira et al., propensity weighted analysis revealed a significant increased risk for residual gastric contents when patients had preoperative digestive symptoms (including nausea/vomiting, dyspepsia, abdominal distension) both in semaglutide users and non-users [40]. There was one reported case (0.24%) of pulmonary aspiration under deep sedation in the GLP-1 RA group; however, it was not reported if this patient had gastrointestinal symptoms prior to his procedure and it was noted that he had confounding factors including history of gastric bypass. Thus, this study by Silveira et al. suggests that pre-endoscopy digestive symptoms may be helpful in identifying higher risk patients, particularly those using GLP-1 RAs. Interestingly, patients undergoing both EGD and colonoscopy showed a protective effect against residual gastric content, possibly secondary to the prolonged (for as long as a day or two prior to the procedures) clear liquid colon preparations prescribed for patients undergoing colonoscopies [40]. This has caused some institutions, including the authors’ own institution, to recommend consideration of a clear liquid diet for 24 h prior to scheduled arrival to the hospital for all patients prescribed GLP-1 RAs. In the four case series and five case reports where gastrointestinal symptoms were evaluated, there was not a consistent relationship between gastrointestinal symptoms and gastric residuals or regurgitant and aspiration events, as detailed in Supplementary Text S2. The case series and report studies where gastrointestinal symptoms were evaluated had patients with confounding factors for increased gastric content, residual food content, and aspiration.

Many major organizations, including the American Society of Anesthesiology (ASA) and the American Gastroenterological Association (AGA), recognize that gastrointestinal symptoms can significantly influence anesthesia management. This can lead to the cancellation or postponement of procedures, the withholding of certain medications, the use of gastric ultrasound for additional evaluation, and decisions regarding the type and induction of anesthesia [14,22,23]. More prospective and retrospective studies are needed to clarify the role of gastrointestinal symptoms, both in general and specifically in patients receiving GLP-1 RAs, in relation to the risk of retained gastric contents.

### 4.5. Holding of Medications Prior to Procedure

There is significant variation in the guidelines and recommendations from major societies regarding the withholding of GLP-1 RAs before a procedure. For example, the ASA advises that patients should pause weekly injections of GLP-1 RAs for one week prior to the procedure and refrain from taking daily oral doses on the day of the procedure. In contrast, several other organizations, including the Association of Anesthetists of Great Britain and Ireland (AAGB) and Centre for Perioperative Care (CPOC), recommend that patients do not need to hold GLP-1 RAs before the procedure [14,15,16,17,18,19,20,23,24]. The AGA recommends an individualized approach regarding the withholding of doses before endoscopy for patients taking GLP-1 RAs solely for weight loss [22]. While this practice may carry little risk, there is uncertainty about its effectiveness in restoring normal gastric motility, so it should not be considered mandatory or evidence based. For patients using GLP-1 RAs for diabetes management, the AGA notes a lack of compelling evidence to support withholding doses, emphasizing that maintaining proper glycemic control is essential before undergoing sedation, anesthesia, and endoscopy procedures [22]. It is important to note that the half-life these medications varies, from daily for daily oral dosing medications to weekly for weekly injection dosing, leading some to suggest that they should be withheld for at least three half-lives to fully allow the medications to clear. There are no prospective studies examining the impact of withholding GLP1 RAs for a set period of time prior to their procedures. Two of the four prospective studies included in our review specifically mentioned when the GLP-1 RA was last taken. The study by Nakatani et al., which specifically mentioned that the GLP-1 RA liraglutide was taken on the day of the capsule endoscopy, and the Sen et al. study, which mentioned that the majority of all 62 patients taking GLP-1 RAs, with the exception of 7 patients, had taken their GLP-1 RAs semaglutide, dulaglutide, or tirzepatide in the last 7 days, with the remaining 7 patients having withheld their GLP-1 RA for 8–15 days [33,34,35]. In the Sen et al. study, there was no significant association between the duration of withholding GLP-1 RA and prevalence of retained gastric contents [35]. In the Nakatani et al. study, there was no significant difference in the gastric, duodenal, and small intestine transit time after GLP-1 RA use compared to before starting GLP-1 RAs in diabetic patients with neuropathy; however, the gastrointestinal transit time was significantly increased after GLP-1 RA administration compared to before starting the GLP1 analogue liraglutide in diabetic patients without neuropathy [33]. The gastric residue rate was significantly higher following the use of GLP-1 RA compared to baseline in diabetics with and without neuropathy. This suggests that holding GLP-1 RAs in diabetics is likely to reduce the risk for gastric residues in diabetic patients with and without neuropathy but is unlikely to affect gastrointestinal transit times in patients with significant diabetic neuropathy. This is consistent with the guidance provided by major societies such as the ASA, AANA, ISMP, and Columbian Society of Anesthesiology and Reanimation (SCARE), which recommend that GLP-1 RAs be held irrespective of the indication of use [14,15,16,17,18,19,20,21,22,23,28,29,30]. The results by Nakatani et al. may be contrary to guidance provided by major societies such as the Australian and New Zealand College of Anaesthetists (ANZCA), who state that holding GLP-1 RAs does not alter the risk of delayed gastric emptying that is inherently associated with diabetes [14,15,16]. Only one of the six retrospective studies included in our review specifically examined the impact of the timing of when the last GLP-1 RA administration was last taken, which was the study by Silveira et al., which reported a mean GLP-1 RA interruption time of 10 days prior to endoscopy, with patients being instructed to hold the GLP-1 RA for 10–14 days prior, but some were unable to follow the instructions for a variety reasons (i.e., last minute notice to fill case cancelation) [40]. They reported GLP-1 RA users had a significant increase in the residual gastric content (defined in their study as any amount of solid content or >0.8 mL/Kg measured from aspiration/suction canister) compared to non-users, but not the subjective amount of residual gastric content found on EGD by visual estimation (small, medium, large). This may suggest that holding the drug for 10–14 days may not be clinically sufficient if the goal is to reduce the residual gastric content to minimize risk for aspiration; however, as mentioned above, it is unclear whether an amount of 0.8 mL/kg is considered clinically significant particularly when the residual gastric content could not be subjectively visually assessed. Despite the qualitative increase in residual gastric content for GLP-1 RA users, there was only one reported case of pulmonary aspiration (0.24%) in the Silveira et al. study, and the patient had last taken his semaglutide 11 days prior and had a prior history of gastric bypass, a significant confounding factor for aspirations [40]. Thus, withholding the drug for 10–14 days appears to result in minimal risk for aspiration despite increased residual gastric content; there are no prospective or retrospective studies assessing patients who have held the drug for just a week, or even 3–4 weeks as others have suggested. Of the case reports and series, only two studies, by Wilson et al. and Raven et al., had patients where the GLP-1 RAs were held according to the ASA guidelines of at least a week for weekly injectables and on the day of surgery for daily oral GLP-1 RAs [14,46,47]. In the case series by Wilson et al., one of the two patients held their oral semaglutide on the morning of surgery and had multiple episodes of bile-tinted projectile vomiting post-extubation [47]. The confounding factor for this regurgitant event was the type of surgery (thyroidectomy), which is often associated with significant post-operative nausea and vomiting. In the other study, by Raven et al., one of the patients in the case series had an initial upper endoscopy which revealed a stomach full of solid food when the patient did not hold his semaglutide; follow-up upper endoscopy after withholding the patient’s GLP-1 RA for 3 weeks revealed an empty stomach [46]. Of note, this patient had multiple confounding factors, including a history of esophagitis on PPI and hypothyroidism on thyroxine at stable doses. From the studies included in our review, there is limited evidence to guide the optimal duration that the GLP-1 receptor should be held prior to procedures.

Interestingly, while several US-based medical societies recommend withholding GLP-1 RAs in the perioperative period, most international guidelines do not advocate for this practice. This discrepancy likely reflects a more precautionary approach in the US, possibly driven by concerns about medico-legal liability, despite the limited evidence supporting a significant risk of aspiration. As more robust data emerge, there is a need to reevaluate these recommendations and develop a global consensus grounded in high-quality scientific evidence.

### 4.6. Prolonged Fasting to Reduce Retained Gastric Food and Residuals

Increased fasting times for solids and liquids are often used as a strategy to reduce gastric contents and aspiration in patients at increased risk. Currently, the majority of the major societies providing perioperative guidance, including the ASA, Institute for Safe Medication Practices in Canada (ISMP), and American Association of Nurse Anesthesiology (AANA), do not make recommendations for increasing the duration of fasting in patients taking GLP-1 RAs [14,15,16,17,18,19,20,21,22,23,28,29]. The CAS is the only society that recommends a prolonged fasting period as a strategy to minimize the risk of aspiration; however, they do not specify the duration of fasting [19]. All of the studies in this review which reported an association with gastric residual content, high gastric residuals, aspiration, and/or regurgitant events reported fasting times exceeded standard ASA guidelines, as prolonged as 20 h for solids and 18 h for liquids in some studies. This also included the retrospective and prospective studies that had reported fasting times of at least 10 h and median of 14.5 h for solids and clear liquids of 9.3 h. Notably, in the study by Silveira et al. in which patients underwent both upper and lower endoscopies, prolonged fasting appeared to have a protective effect against residual gastric content [40]. Patients undergoing colonoscopies fast for 48 h with solids and 24 h for liquids. A prolonged fast of this magnitude may be associated with a reduced risk for residual gastric contents and aspiration/regurgitant events. Further studies are necessary to determine if and the duration of prolonged fasting reduces retained gastric food contents and residuals, and whether this is procedure-dependent.

### 4.7. Gastric Ultrasound Studies to Assess Aspiration Risk

Gastric ultrasound is a noninvasive tool that can be used to assess aspiration risk and guide clinical management. The CAS, ASA, ANZCA, and AGA all recognize gastric ultrasound as a potentially helpful tool to assess risk and guide perioperative management of patients on GLP-1 RAs [16,17,18,19,22,23]. Only four studies involved gastric ultrasound to assess patients with GLP-1 RAs. These studies included the two prospective studies by Sherwin et al. and Sen et al., the case series by Kittner et al., and the case report by Giron Arango et al. [35,36,45,46,47,48,49,50,51]. The prospective study by Sherwin et al. involved assessment for solid gastric contents using gastric ultrasound [36]. The study found that, after an 8 h fast, 70% of participants in the GLP-1 RA group displayed solid gastric contents when assessed in the supine position, while 90% showed solid contents in the lateral position. In comparison, only 10% and 20% of participants who did not use GLP-1 RAs showed solid gastric contents in the supine and lateral positions, respectively. Subsequently, participants underwent a gastric ultrasound study two hours after consuming clear liquids. The results showed no difference in gastric content in the lateral position; however, a significant difference was observed in the supine position, where 90% of the control group had empty stomachs compared to only 30% of the GLP-1 RA group. Of note, patients with medical conditions associated with delayed gastric emptying were not excluded; participants were noted to have a history of diabetes, Parkinson’s disease, multiple sclerosis, amyloidosis, and scleroderma, all of which are associated with delayed gastric emptying. The authors also noted in their study that they have data from non-orthopedic patients who underwent gastric ultrasound during the same period at their institution and revealed seven out of eight patients on GLP-1 RAs with adequate fasting periods had retained solid food. The prospective study by Sen et al. reported higher retained gastric contents in the GLP-1 RA group compared to the non-user group (56% versus 19%) evaluated by preprocedural gastric ultrasound; of note, the patients had confounding factors, including diabetes, GERD, home opioid use, and high ASA physical status classification [35]. The case series by Kittner et al. reported retained solids in all three patients who had fasted 10–14 h and were assessed preoperatively for orthopedic procedures who had fasted 10–14 h, resulting in case postponements; of note, all three patients had type 2 diabetes [45]. The case report by Giron-Arango et al. revealed increased gastric volume assessed by gastric ultrasound consistent with a full stomach; this patient also had type 2 diabetes, which may have been a confounding factor [51]. Further studies are necessary to determine the utility of gastric ultrasound in improving clinical outcomes.

### 4.8. Type, Dosing, and Duration of GLP-1 RA Use

There is significant concern in the literature that differences in the type, dosing, and duration of action of individual GLP-1 RAs may be important variables impacting gastric emptying, regurgitation, and aspiration risk. Specifically, higher doses of medications may result in a significant slowing of gastric emptying compared to lower dosing of medications using indirect measurements of gastric emptying, and that the duration of GLP-1 RA use may play a role given studies suggesting tachyphylaxis after prolonged GLP-1 RA use [63,64,65,66]. None of the major societies provide guidance based on the type, dosing, and duration of GLP-1 RA aside from the duration of time that individual GLP-1 RA drugs should be held based on their duration of action [14]. From the studies included in this review, there is insufficient evidence to indicate a clear association between the type, dose, and duration of GLP-1 RA use and gastric food content, increased gastric residuals, aspiration, and/or regurgitant events. The study by Kobori et al. found that gastric residue occurrence was highest with semaglutide 1.0 mg oral dosing; of note, patients in the study took semaglutide injection doses ranging from 0.25 to 1.0 mg [39]. The study also found no gastric residue in patients treated with doses of liraglutide (≤1.5 mg), lower doses of semaglutide (0.25 mg), and any dose of oral semaglutide, lixisenatide, or exenatide. In contrast, many of the other studies showed incidences of gastric food content, increased gastric residuals, aspiration, and/or regurgitant events with these formulations and dosing. The studies included in this review had varying reports of GLP-1 RA use duration. Gastric food content increased gastric residuals, aspiration, and/or regurgitant events occurring during both shorter- and longer-term use of GLP-1 RA in the studies reviewed. In their retrospective study, Silveira et al. report that the duration of preoperative semaglutide cessation did not impact the presence or absence of residual gastric contents [40].

Nevertheless, the ASA, AANA, and ISMP provide specific guidance on the duration of withholding GLP-1 RAs beyond just the day of surgery [23,28,29]. Both the ASA and AANA suggest that for weekly dosing of GLP-1 RAs, consideration is to be given to withholding the medication a week before surgery; for GLP-1 RAs that are taken daily, consideration should be given to withholding on the day of surgery [23,24,25,26,27,28]. The ISMP suggests that consideration be given to withholding GLP-1 RAs for at least three half-lives before the procedure [29]. The ASA advises that GLP-1 RAs be withheld before surgery/procedures, regardless of the indication, dose, or type of procedure [23]. The ASA also notes that the effects on gastric emptying decrease with long-term use, likely due to tachyphylaxis from vagal nerve activation, but does not provide any details related to specific adjustments in management regarding the duration of GLP-1 RA use [23]. 

### 4.9. Limitations

There are limitations with our scoping review of the literature. Many of the studies included in our review are of low-quality evidence, including the case reports and series. The higher-quality studies, both retrospective and prospective, were often limited by small sample sizes, single institutions, and lack of blinding. Many of the studies included incomplete or the absence of details related to GLP-1 RA use (i.e., specific type, dosage, frequency, duration, timing of discontinuation), patient medical and surgical history, other potential confounding factors, and other notable findings (i.e., gastrointestinal symptoms, elevated gastric residual volume, residual gastric food contents) related to GLP-1 RA use. Finally, our review included only studies published in English and did not examine those found in the gray literature, such as technical reports and conference proceedings.

## 5. Conclusions

Our scoping review is the first to provide the most comprehensive review of the available literature regarding GLP-1 receptor agonists and their associated risk for residual food content, increased gastric residuals, aspiration, and/or regurgitant events. There is significant variability in the findings reported, and many of these studies contain confounding factors that may impact the findings of an association of GLP-1 receptor agonists. While GLP-1 RAs do increase residual gastric contents in line with their mechanism of action, the currently available data do not suggest a significant increase in aspiration events associated with their use and the withholding of GLP-1 RAs to reduce aspiration events, as is currently recommended by many major societal guidelines. Large RCTs may be helpful in further elucidating the impact of GLP-1 RAs on perioperative aspiration risk.

Importantly, withholding GLP-1 RAs in the perioperative period may lead to significant elevations in blood glucose, which introduces its own risks, such as impaired wound healing and an increased likelihood of surgical site infections. Therefore, careful consideration of both glycemic control and aspiration risk is critical. Future guidelines should strive to balance these competing risks, developing evidence-based recommendations that prioritize overall patient safety and outcomes.

## Figures and Tables

**Table 1:** Summary of studies with reported endoscopic, ultrasound, and nasogastric evaluation for increased residual gastric volume retained food contents, as well as incidences of regurgitation and aspiration events.

Author, Year, and Journal	Type of Study	How Many Patients, Age	BMI	Confounding Factors and Exclusion Criteria	Medication Dosing, Frequency, and Duration of Use	Indication for Medication	Fasting Time Prior to Procedure	Holding GLP-1 Agonist Duration	Symptoms on Presentation	EGD, Capsule, Ultrasound, or Gastric Volume Findings, and Other Notable Imaging Findings and Outcomes	Aspiration or Regurgitant Event and Outcomes
Nakatani et al. Diabetes Metab. 2017 [33]	Prospective observational study	15 patients (mean age 60.0 ± 13.6 and 71% male)	BMI 26.9 ± 3.8	Type 2 diabetes Exclusion criteria: Type 1 diabetes, Type 2 diabetes on insulin, history of arrhythmias and treatment, marked dysautonomia, history of pancreatitis, ileus, or abdominal surgery, and contraindication to capsule endoscopy	Liraglutide 0.3 mg initially and then titrated up by 0.3 mg weekly to a final dose of 0.9 mg weekly, total duration of drug for 1 month prior to capsule endoscopy	Diabetes	11 h	Taken day of capsule endoscopy	Symptoms not mentioned for day of capsule endoscopy procedure. Reported that the digestive symptoms induced by liraglutide, such as nausea and diarrhea, were not severe, and did not require the drug to be discontinued in any patients.	Capsule endoscopy was performed to study the effects of liraglutide on GI motility in diabetic patients, comparing those with and without diabetic neuropathy (DN). Capsule endoscopy was performed both prior to start liraglutide and after one month of liraglutide administration. In diabetic neuropathy (DN) patients, there was no significant difference in the gastric time after compared to before starting liraglutide (0:48:40 ± 0:32:52 h vs. 1:12:36 ± 1:04:30 h, *p* = 0.19). In non-DN patients, gastric time was significantly increased after liraglutide administration compared to before starting liraglutide (2:33:29 ± 1:37:24 h vs. 1:01:30 ± 0:52:59 h, *p* = 0.03). Duodenal and small intestine transit times also increased significantly in only the non-DN group (6:45:31 ± 2:41:36 vs. 3:51:03 ± 0:53:47 h, *p* = 0.03) and not the DN (6:38:42 ± 3:52:42 h vs. 4:10:34 ± 0:25:54 h, *p* = 0.09) group. GI residue rates significantly increased post-liraglutide compared to prior to starting liraglutide in both the DN (90.0 ± 9.1% vs. 32.1 ± 24%, *p* < 0.001) and non-DN (78.3 ± 23.9% vs. 32.1 ± 35.3%, *p* < 0.001) groups. The findings suggest liraglutide slows gastric emptying and GI motility, with effects less pronounced in DN patients, likely due to dysautonomia.	Not applicable
Quast et al. Diabetes Care. 2020 [34]	Randomized, bicentric, investigator-blinded, parallel group study	57 patients (mean age 60.7 yo ± 7.6 and 41.7% female in lixisenatide group, 60.2 yo ± 7.3 and 30.8% female in liraglutide group)	BMI 18–40, mean baseline BMI of 32 and 31.4 in lixisenatide and liraglutide treatment groups, respectively	Type 2 diabetes, DPP-IV inhibitor medications part of some patients’ medication regimen. Exclusion criteria included patients with decompensated diabetes (A1c > 10), preexisting concomitant diseases not typically associated with diabetes (i.e., liver, renal, and hepatic disease), contraindications to GLP-1 RAs and DDP-IV inhibitors, use of medications that impact gastrointestinal motility and secretions, history of GERD or overt gastroparesis, smokers.	Lixisenatide 10 mcg once daily for 1 week, followed by 20 mcg once daily for remainder of the trial; total duration 10 weeks Liraglutide 0.6 mg once daily for week 1, followed by 1.2 mg once daily for week 2, followed by 1.8 mg once daily for the remainder of the trial; total duration 10 weeks	Type 2 diabetes	Duration not specified; mentions that fasting started 8 pm day prior to tests	Not mentioned	Heartburn, nausea, vomiting, diarrhea or loose stools and hypoglycemic events were reported for patients treated with lixisenatide and liraglutide, but no difference between the two groups		Not specifically mentioned related to anesthesia procedures. Esophageal pH and manometry revealed no significant difference in reflux episode frequency and severity, as well as esophageal motility and lower esophageal sphincter functionality compared to baseline. Gastric acidity was significantly reduced by 20.7% (−40.6, −0.8) (*p* = 0.041) with GLP-1 RAs. The octanoate acid breath test revealed lixisenatide significantly increased the gastric emptying half-time by 52 min (95% CI 16 to 88, *p* = 0.0065) and liraglutide by 25 min (CI 3 to 46, *p* = 0.025).
Sherwin et al. Can J Anesth. 2023 [36]	Prospective study	20 volunteer participants (10 taking semaglutide, 10 controls) (median age 41.5 yo and 70% male in semaglutide group, 31.5 yo and 50% male in control group)	BMI all < 30, higher in semaglutide group compared to control group (median, 26.9 vs. 20.5, *p* < 0.001)	All but one participant taking semaglutide for weight loss; one was taking semaglutide for diabetes. No patients in control group had diabetes. Other medical conditions associated with delayed gastric emptying were not excluded. Included in the study were patients with diabetes, Parkinson’s disease, multiple sclerosis, amyloidosis, and scleroderma. Exclusion criteria included past history of upper abdominal surgery, hiatal hernia, and currently taking medications associated with delayed gastric emptying (i.e., opioids, proton pump inhibitors, tricyclic antidepressants, calcium channel blockers, antipsychotic drugs, or lithium).	Semaglutide; dose 0.25 mg (50%), 0.5 (40%), and 0.75 (10%); frequency daily (10%) and weekly (90%); duration on therapy 1–2 weeks (40%), 3–4 weeks (40%), 5–8 weeks (!0%), >8 (10%) Majority on <4 weeks of therapy at time of study enrollment	Indication for all patients was weight loss, except one patient was taking for diabetes.	>10 h for all participants	Not mentioned	Gastrointestinal symptoms not evaluated	This prospective study evaluated the effect of GLP-1 receptor agonists (GLP-1RAs) on gastric emptying using ultrasound. After an eight-hour fast, 70% of participants on semaglutide showed solid gastric contents in the supine position, compared to 10% of controls (risk ratio [RR] = 3.50, 95% CI 1.26–9.65, *p* = 0.02). In the lateral position, these numbers were 90% for semaglutide users vs. 20% for controls (RR 7.36, 95% CI 1.13- 47.7, *p* = 0.005). Participants subsequently had a gastric ultrasound study performed two hours after clear liquid intake. No lateral position differences were noted, but in the supine position, 90% of controls had an empty stomach versus 30% of the semaglutide group (*p* = 0.02). The semaglutide group’s gastric solids had a layered/yogurt-like consistency on gastric ultrasound imaging. These findings suggest GLP-1RAs may slow gastric emptying, potentially increasing aspiration risk during anesthesia despite adequate fasting.	Not mentioned
Sen et al. JAMA Surgery. 2024 [35]	Propspective cros-sectional study	124 patients (median age 56 yo [IQR, 46–65 yo], 60% female), of which 50% (62 patients) were taking once-weekly GLP-1 RA and the remainder 50% were non-users. GLP-1 RA group: median age 59 yo [IQR, 48–65 yo], 60% female; Control group: median age 53 yo [IQR, 43–64 yo], 61% female	Median BMI 33.9 [IQR, 30.7–39.2]	Diabetes, GERD, home opioid use, pain, ASA physical status classification. Exclusion criteria included patients with altered gastric anatomy (i.e., prior gastric surgery), pregnancy, recent trauma (<1 month), or inability to lie in the right lateral decubitus position for gastric ultrasound.	Semaglutide, dulaglutide, tirzepatide	Not mentioned	Standard fasting guidelines	Of 62 patient taking GLP-1 RAs, all but 7 patients took their GLP-1 RA in the last 7 days. The remainder of the patients had withheld their GLP-1 RA for 8–15 days.	Not mentioned	Retained gastric content was higher in the GLP-1 RA compared to the non-user group (56% versus 19%) evaluated by preprocedural gastric ultrasound. There was no significant association between the duration of withholding GLP-1 RA and prevalence of retained gastric contents.	Not mentioned
Anazco et al. Clinical Gastroenterology and Hepatology. 2023 [37]	Retrospective, multiple hospital cohort study	2968 unique patients taking GLP-1 RA prescribed prior to endoscopies totaling 4134 endoscopic procedures (average age and sex distribution not mentioned for cohort)	Average BMI of patients not mentioned	Included all patients who had been prescribed any injectable GLP-1 RA and had a prior upper endoscopy after initiation of GLP-1 RA. History of patients who aspirated included ASA III, diabetes, and/or GERD. Excluded patients who had aspirations mentioned in clinical notes prior to the first upper endoscopy event.	Not specifically mentioned. The patients initially identified as having GLP-1 RA prescriptions (irrespective of endoscopy) for the following medications: lixisenatide, tirzepatide, exenatide, liraglutide, dulaglutide, semaglutide (dosing, frequency, duration).	Not mentioned	Not mentioned	Not mentioned	Not mentioned	Not rigorously assessed except in the context of 2 definite pulmonary aspiration events from 4134 endoscopic procedures reviewed after GLP-1 RA prescription using a previously validated automatic search algorithm of the electronic medical record system. One of the two patients who had an aspiration event had reported retained food content in stomach and duodenum during upper endoscopy performed under MAC. The other patient who had an aspiration rate was noted to have ectasia with bleeding and underwent argon plasma coagulation (APC).	Using a previously validated automatic search algorithm of the electronic medical record system, the authors identified 2 definite episodes of pulmonary aspirations from 4134 endoscopic procedures. The aspiration rate was 4.8 cases per 10,000 endoscopies in comparison to a prior retrospective study from the institution which revealed an aspiration rate of 4.6 cases per 10,000 endoscopies previously using the same automatic search algorithm. They defined an aspiration event as presence of bilious material or particulate matter in the airway during visualization, and/or radiographic evidence of post-procedural pulmonary infiltrates. The 2 aspiration events occurred in one patient who was a 47 year old female, ASA III, with normal weight, on dulaglutide (3.0 mg weekly, started 30 months ago) for diabetes (HbA1c 6.7) that had an upper endoscopy for abdominal pain and diarrhea to rule out celiac disease, was found to have retained food contents in stomach and duodenum during upper endoscopy, had massive vomiting upon scope removal with direct visualization of aspirated contents in the airways and chest imaging compatible with aspiration, and required intubation and ICU admission for 3 days, with vasopressors, and was discharged from the hospital after 5 days with oxygen; the other patient was a 72 year old female, ASA III, with obesity (class II) and GERD, taking semaglutide (0.5 mg weekly, started 3 months ago) for obesity and no symptoms on initiation of GLP-1 RA, had presented for upper endoscopy for iron deficiency anemia and possible GAVE, found to have diffuse gastric antral vascular actasia and bleeding requiring APC, did not have visualization of retained food contents in stomach and duodenum, had persistent hypoxemia after the procedure with imaging compatible with aspiration, not requiring ICU admission, and was discharged home after 8 days of hospitalization.
Bi et al. Digestive Diseases and Sciences. 2021 [38]	Retrospective study	2150 patients (mean age 55 yo ± 16; 67% female)	BMI not noted	Medications known to impair gastric antral motility and/or cause delayed gastric emptying (GE) including GLP-1 agonists, opioids, antiacids (i.e., histamine receptor antagonists and PPIs), cardiovascular medications (i.e., beta and calcium channel blockers), and others. Patients on GLP-1 agonists represented a small fractions of patients, with exact number not reported. Of note, patients with diabetes were excluded from GLP-1 RA and retained gastric food analysis Excluded patients who had neither structural foregut abnormalities nor medical comorbidities associated with retained gastric food in the multivariate analysis of prevalence (i.e., diabetes, amyloidosis, or a diagnosis of gastroparesis)	Not mentioned	Not specifically mentioned. Of note, patients with diabetes were excluded from GLP-1 RA and retained gastric food analysis.	Not mentioned; assumed adherence to standard pre-procedural fasting (6–8 h)	Not mentioned	Not mentioned. Study included patients presenting to endoscopy unit for any indication.	GLP-1 RAs were not found to have a statistically significant association with retained gastric food (RGF) during EGD on multivariate analysis, although they showed significance on univariate analysis. However, due to the small number of patients taking GLP-1 agonists in the study, the odds ratio did not reach statistical significance, suggesting a potential association that warrants further investigation. Opioids, antacids, and cardiovascular medications were associated with RGF on univariate and multivariate analysis.	Not evaluated
Kobori et al. 2023. Journal of Diabetes Investigation [39]	Matched pair case-control study	1128 individuals with diabetes, had EGD performed, and taking GLP-1 RA matched against control group not taking GLP-1 RA over a 2-year period (median age 70 [IQR 62–76] for GLP RA group, 72 yo yo [IQR 63–77] for non-GLP-1 RA group, approximately 80% male in both groups)	Not mentioned	Diabetes (median HbA1c was 7.3) Excluded patients with history of esophageal or gastric operation, unable to fast and no A1c within 3 months prior to EGD Patients unable to fast were also excluded.	Dulaglutide 0.75 mg, liraglutide <0.3 to 1.8 mg, semaglutide 0.25–1.0 mg, oral semaglutide 3–7 mg, lixisenatide <10–20 ug, exenatide 20 ug (frequency and duration note noted)	Not specifically mentioned	>=12 h	Not specifically mentioned	Not mentioned; indications for EGD procedures not mentioned	The occurrence of gastric residue was significantly higher in those receiving GLP-1 RA treatment (5.4% vs. 0.49%, *p* = 0.004). The specific GLP-1 RAs used among the 11 patients with gastric residue included liraglutide (1.8 mg daily, 2 of 19 patients or 10.5%), dulaglutide (0.75 mg weekly, 5 of 90 patients or 5.6%), and semaglutide (0.5 mg weekly, 2 of 17 patients or 11.8%; 1.0 mg weekly, 2 of 9 patients or 22.2%). Patients with gastric residue were notably younger than those without. The distribution of GLP-1 RA treatments among these patients indicated varying percentages of gastric residue occurrence, with semaglutide 1.0 mg weekly showing the highest rate (22.2%) among the specific doses and types of GLP-1 RAs mentioned. No gastric residue was reported in patients treated with lower doses of liraglutide (≤1.5 mg), lower doses of semaglutide (0.25 mg), any dose of oral semaglutide, lixisenatide, or exenatide.	Not mentioned
Silveira et al. Journal of Clinical Anesthesia. 2023 [40]	Retrospective observational study	404 patients (33 in semglutide group, 371 in non-semaglutide group) (median age 50.8 (percentile 25–75% 39–64) and 48.5% female)	Obesity (BMI > 30) observed in 19.9% patients, median (percentile 25–75%) BMI was 26.2 (22.98–28.73)	Patients with medical issues included diabetes, PPI use, prior abdominal surgeries, treated hypothyroidism, psychiatric illness, and ASA 1–3. Included patients presenting for colonscopy in addition to EGD, as well as just EGD. Exclusion criteria included conditions such as gastric outlet obstruction, gastrointestinal mechanical obstruction, active gastrointestinal bleeding, recent abdominal surgery within 2 months, emergency procedures, ASA-PS >= IV, ICU admission, as well as other medical conditions (i.e., chronic renal and liver disease, pregnancy, chronic opioid use, etc) and the use of medications (i.e., tricyclic antidepressants, opioids, prokinetics, histamine H2-receptor antagonists) known to affect gastric emptying, except for semaglutide. Patients using GLP-1 agonists other than semaglutide were also excluded.	Semaglutide (dose, frequency, and duration not reported)	Primary indication for weight loss (87.8%), followed by diabetes (12.2%)	Median fasting (percentile 25–75%) for clear fluids 9.3 (5.0–12.8) hours and 14.5 (12.2–28.7) hours for solids.	Patients instructed to stop 10–14 days prior to elective procedure but some unable to follow instructions for a variety of reasons (i.e., last minute notice to fill case cancellations); mean interruption time approximately 10 days	Semalutide group: 27.3% with digestive symptoms; non-semaglutide group: 4.5% with digestive symptoms	The retrospective study of patients undergoing upper endoscopies found that semaglutide use perioperatively significantly increased residual gastric content (RGC) (defined as any amount of solid content, or >0.8 mL/Kg measured from aspiration/suction canister) compared to non-users (24.2% vs. 5.1%, *p* < 0.001), but not the subjective amount of RGC found on EGD by visual estimation (small, medium, large). Propensity weighted analysis revealed increased risk for RGC with factors such as the use of semaglutides (Prevalence Ratio (PR) = 5.15, 95% CI (1.92–12.92), *p* < 0.001), preoperative digestive symptoms (including nausea/vomiting, dyspepsia, abdominal distension) (PR = 3.56. 95% CI 2.25–5.78, *p* < 0.001), semaglutide use and digestive symptoms (PR = 16.5, 95% CI 9.08–34.91, *p* < 0.001), semaglutide use and no digestive symptoms (PR = 9.68, 95% CI 5.6–17.66, *p* < 0.001), and no semaglutide use and digestive symptoms (PR = 4.94, 95% CI 1.32–15.77, *p* = 0.0098); while patients undergoing both EGD and colonoscopy showed a protective effect against RGC (PR = 0.25, 95% CI 0.16–0.39, *p* < 0.001). Pre-endoscopy digestive symptoms were associated with increased RGC, suggesting their relevance in identifying higher risk patients, especially those using semaglutide perioperatively. The duration of preoperative semaglutide cessation did not impact the presence or absence of RGC (10.5 ± 5.5 and 10.2 ± 5.6 days, *p* = 0.54).	There was one reported case (0.24%) of pulmonary aspiration under deep sedation for upper endoscopy in a 63-year-old man with obesity (BMI 37.7), prior history of gastric bypass, and semaglutide use (last taken 11 days prior) who had adequately fasted (12.4 h for solids and clear fluids) and reported no digestive symptoms pre-procedure. No negative sequelae were specifically reported for this aspiration event. Of note, their institutional practice is to stop the procedure or intubate to prevent aspiration if excessive gastric residue is found.
Stark et al. Annals of Pharmacotherapy. 2021 [41]	Retrospective cohort study with matched controls	59 patients prescribed GLP-1 RAs over 5 year period, January 2015, matched 1:2 against 118 control group for diagnosis of diabetes and/or cirrhosis given their effects on delayed gastric emptying (mean age 64 yo ± 10 and 83% males for GLP-1 RAs, 66 yo ± 10.2 and 94% males for matched controls)	Mean BMI of 33 for both groups	Diabetes, cirrhosis, concomitant medications that slow GI transit, concomitant prokinetic medications, GERD, Barrett’s esophagus, dysphagia, and gastric polyps. Excluded patients with bowel obstruction; prior diagnosis of gastroparesis; history of esophageal, gastric, or thoracic surgery; or if the indication for EGD was for food impaction, foreign body, or active GI bleed.	Duglatide, liraglutide, exenatide, semaglutide (dose, frequency, and duration of use not specified)	Does not mention specifically	Not mentioned	Not mentioned	Not specifically mentioned. Indications for EGD procedures included anemia, GERD, Barrett’s esophagus, dysphagia, abdominal pain, history of gastric polyps, surgical screening, and other.	The primary endpoint, the odds of observing retained food content during EGD, showed a higher, yet not statistically significant, incidence in the treatment group compared to the control (6.8% vs. 1.7%; Odds Ratio [OR] 4.22, 95% Confidence Interval [CI] 0.87–20.34). Specifically, cases of retained food involved patients on either dulaglutide or liraglutide therapy (two instances noted for each medication). Secondary endpoints evaluated the necessity for gastric lavage or a repeat EGD due to inadequate visualization. Both groups had a single instance of gastric lavage (1.7% vs.0.8%, *p* = 0.62), which removed retained food to achieve satisfactory visibility. No instances necessitating a repeat EGD were recorded.	Not mentioned
Wu et al. CJA. 2024 [42]	Retrospective cohort study	GLP-1 RA group: 64 patients; Control (patients started on GLP-1 RA within 1000 days after EGD): 69 patients GLP-1 RA group: median age 64.1 yo [IQR, 56.6–68.9], 62% female; Control group: median age 58.5 yo [IQR, 45.7–67.6 yo], 53% female	GLP-1 RA group: median BMI 34.0 [IQR, 30.0–38.9]; Control group: median BMI 34.0 [IQR, 30.0–37.6]	Incidence of diabetes (96% GLP-1 RA vs. 25% control), insulin use (31% GLP-1 RA vs. 2% control), oral diabetes medications (41% GLP-1 RA vs. 22% control), nausea on presentation (12% GLP-1 RA vs. 2% control), ASA status III or IV (81% GLP-1 RA vs. 57% control)	In GLP-1 RA group: 70 procedures performed in patients taking semaglutide, 11 liraglutide, 6 dulaglutide, 1 tirzepatide, and 2 combination of two different drugs	Not mentioned	GLP-1 RA group: median 16 h [IQR, 14–19]; Control group: median 16 h [IQR, 14–19 h]	Not mentioned; of note, no instructions to withhold GLP-1 Ras prior to the procedure	Nausea on presentation in 12% GLP-1 RA vs. 2% control group patients	Visible gastric content on EGD was reported in 17 cases (19%) in GLP-1 RA group and 5 cases (5%) in control group (*p* = 0.004). In GLP RA group, 4 of 71 procedures that started off as MAC, residual gastric contents resulted in emergent endotracheal intubation, and in one case a pulmonary aspiration event which resulted in transfer to ICU; extubated 4 h later and discharged the next day. In the non-user group, there were no such reported events.	Pulmonary aspiration reported in 1 case in GLP-1 RA group (total of 90 procedures from 64 patients) and none in control group (total of 102 procedures from 69 patients). In the one case of a pulmonary aspiration event previously reported in Klein et al., the patient was emergently intubated, had bronchoscopy performed which revealed food remains in the trachea and bronchi, was transferred to the ICU, extubated 4 h later, and discharged the next day.
Avraham et al. Anaesthesia Rports. 2024 [43]	Case Series	2 (Patient 1: 70 yo M) Patient 2: 25 yo F)	Patient 1: BMI 35 Patient 2: BMI 32	Patient 1: Type 2 diabetes (HbA1c 7%) Patient 2: none reported	Patient 1: semaglutide 1 mg weekly subcutaneous injections Patient 2: semaglutide 1 mg weekly subcutaneous injections	Patient 1: Not mentioned Patient 2: weight loss	Patient 1: 12 h Patient 2: >8 h	Patient 1: 6 days prior to procedure Patient 2: 4 days prior to procedure	Patient 1: No nausea and vomiting Patient 2: Not mentioned	Patient 1: No mention of presence or absence of residual gastric contents on EGD for ERCP following regurgitant episode during laryngoscopy. Nasogastric suctioning performed following regurgitatn epsidoe with no gastric residual volume reported Patient 2: Nasogastric suctioning performed following regurgitant episode following LMA removal following completion of surgery with no gastric residual volume reported.	Patient 1: During laryngosocopy following RSI for ERCP, large volume of regurgitant contents with particulate food noted. CXR with bilateral infiltrates. Patient transferred to ICU following procedure and extubated the following day, and was discharged from hospital after a week. Patient 2: Regurgitation of solid and liquids following LMA removal after completion of surgery for I&D of breast abscess. Patient head tilted and sucitoned, rapid sequence intubation and then extubated awake. Transferred to PACU, with reportedly normal chest xray and observation that was normal.
Kalas et al. J Investig Med High Impact Case Rep. 2021 [44]	Case Series	2 patients (Patient 1: 52 yo F; Patient 2: 57 yo F)	Not mentioned	Patient 1: Type 2 diabetes (A1c 5.7), medications including PPIs and antispasmodics (anticholinergics) (not clear from report if taking at the time) Patient 2: Type 2 diabetes (A1c 8.2)	Kalas et al.: Patient 1: semaglutide subcutaneous weekly (dose not mentioned), unclear duration relative to intial endoscopy procedure; Patient 2: dulaglutide subcutaneous weekly (dose not mentioned), unclear duration relative to initial endoscopy procedure	Not mentioned for both cases	Not mentioned for both cases	Not mentioned for both cases	Patient 1: postprandial epigastric pain, fullness, bloating, and nausea Patient 2: abdominal bloating, nausea, and vomiting	Patient 1: Prior upper and lower endoscopies negative for obstruction. CT abdomen and HIDA scan and abdominal doppler blood flow study unremarkable. Initial scintigraphic GES revealed delayed gastric emptying with 24% isotope retention at 4 h (normal residual <10%). Semaglutide was held for 6 weeks, leading to symptom resolution and a repeat GES showing normalization of gastric emptying. Patient 2: Prior upper and lower endoscopies no obstruction or other abnormalities. A 4 h GES showed 35% isotope retention at 4 h, indicating delayed gastric emptying. Dulaglutide was stopped for 4 weeks, resulting in gradual symptom resolution. A subsequent GES confirmed normal gastric emptying.	Not mentioned for both patients
Kittner et al. The Journal of Bone and Joint Surgery. 2023. [45]	Case Series	3 patients (Patient 1: 75 yo M; Patient 2: 72 yo F; Patient 3: 61 yo M)	All 3 patients obese, BMI not noted	All patients had Type 2 diabetes (A1c not noted)	Semaglutide (dose, frequency, and duration not mentioned)	Not mentioned. Of note, all patients were obese and had diabetes.	Patient 1: 11 h Patient 2: 10 h Patient 3: 14 h	Case 1: last taken day before surgery Case 2: last taken 6 days prior to surgery Case 3: last taken day before surgery	All patients noted new GI symptoms since starting GLP-1 RAs	Gastric ultrasound revealed retained solids for all three patients preoperatively who were presenting for orthopedic procedures, resulting in case postponement. Of note, authors note that this article does not include data on non-orthopedic patients who underwent gastric ultrasound during the same timeframe at their institution and revealed 7 out of 8 patients (87.5%) on GLP-1 RAs with adequate fasting periods who had retained solid food.	No aspiration events; cases postponed after gastric ultrasound revealed retained solids.
Raven et al. Am J Med. 2023 [46]	Clinical communication to the editor on case series	2 patients (Patient 1: 62 yo M; Patient 2: 61 yo M)	Both patients with BMI 30	Patient 1: history of esophagitis on PPI and hypothyroidism on thyroxine at stable doses Patient 2: history of GERD, HIV on HAART	Patient 1: semaglutide 1 mg (frequency unknown), duration unclear but mentioned it was started week prior to procedure Patient 2: liraglutide daily subcutaneous (dose and duration unknown)	Not mentioned	Patient 1: 13 h for first EGD, 12 h for repeat EGD Patient 2: fasted 10 h	Patient 1: no withholding for 1st EGD (had started week prior to procedure), withheld for 3 weeks prior to 2nd EGD Patient 2: not mentioned	Patient 1: None specifically mentioned. Indication for EGD for 1st patient was routine follow-up for esophagitis. Patient 2: indication for EGD was investigation of reflux	Patient 1: Initial EGD revealed stomach full of solid full and procedure aborted. Repeat EGD when semaglutide held for 3 weeks revealed empty stomach after 12 h of fasting. Patient 2: EGD found stomach full of solid food, and procedure aborted.	Not specifically mentioned. EGD procedures aborted when EGD found stomach full of solid food for both patients.
Wilson et al. A&A Practice. 2023 [47]	Case series	2 (Patien1: 55 yo F; Patient 2: 34 yo F)	Patient 1: BMI 48.84 Patient 2: BMI 50.1	Patient 1: Type 2 diabetes (no A1c reported), ketamine used for sedation (associated with increased secretions) Patient 2: Type 2 diabetes (A1c 5.9), asymptomatic GERD, thyroid surgery (given post-op regurgitation)	Patient 1: dulaglutide 1.5 mg subcutaneously weekly (duration not specified) Patient 2: semaglutide 7 mg oral daily (duration not specified)	Both patients: diabetes	Patient 1: solids for 10 solids, clear liquids for 4 h Patient 2: solids for 16 h, clear liquids for 5 h	Patient 1: Taken her dulaglutide injection in the past week Patient 2: Held oral semaglutide on morning of surgery	Both patients: no symptoms of gastroparesis, no abdominal surgeries, or abnormalities	None	Patient 1: Developed oropharyngeal secretions and labored respirations after regional anesthesia and initiating monitored anesthesia care with fentanyl, midazolam, propofol, and ketamine for foot arthrodesis, prompting conversion to general anesthesia. Upon intubation, bilious particulate matter was suctioned from the oropharynx, and gastric contents were evacuated. No respiratory symptoms post-procedure were reported, and patient was discharged after 2 h in recovery. Patient 2: Not specifically mentioned. After thyroidectomy under general anesthesia, the patient after extubation experienced multiple episodes of projectile vomiting with bile-tinted particulate matter. Lung auscultation was unremarkable, and the patient received supportive care with suctioning and oxygen therapy, and was discharged the following day per protocol for thyroidectomy procedures.
Almustanyir et al. Cureus. 2020 [48]	Case report	1 (18 yo F)	BMI 31.9	Type 2 diabetes (A1c 8.4)	Liraglutide (dose, route, frequency, and duration of use not specified)	Diabetes and obesity	Not mentioned	Not mentioned	Gastroparesis symptoms after initial dose of liraglutide	EGD with no proof of an obstructing lesion X-ray with non-obstructive gas pattern with intestinal dilation. CT abdomen with isolated moderate-grade gastric distention, with smooth, thin wall and no evidence of distal gastric, pyloric, or proximal duodenal obstructing masses. Gastric suctioning through NGT and discontinuation of liraglutide and short regimen of antiemetics resulted in symptom resolution.	Not mentioned
Espinoza et al. J ECT. 2024 [49]	Case report	1 (64 yo F)	Class 3 Obesity (BMI >40)	Hypothyroidism	Semaglutide subcutaneously weekly (dosing and duration of use not mentioned)	Obesity	Adequate	Not held, last taken days prior	Not mentioned	Not assessed in patient presenting for ECT	ECT performed after risk/benefit discussion with patient. Uneventful ECT treatment performed with monitored anesthesia care with mask ventilation, without any significant anesthesia modifications. She has subsequently had 9 uneventful ECT treatments with semaglutide held 1 week prior to treatments and resumed after treatments and has had continued weight loss.
Fujino et al. Cureus. 2023. [50]	Case report	1 (31 yo F)	BMI 45.6	Type 2 diabetes (A1 5.9)	Semaglutide 0.25 mg weekly, duration approximately 1 month	Obesity	Solids for 10 h for first endoscope; for second endoscopy, liquid fast for 36 h	Did not hold, taken as prescribed for first endoscopy; for second endoscopy, last taken 7 days prior to procedure (only held on day of procedure)	No symptoms; of note, EGD performed in preparation for bariatric surgery	EGD initially performed in preparation for bariatric surgery revealed food residue in the stomach, leading to procedure being aborted to minimize risk for aspiration. A month later, repeat EGD performed after 36 h liquid diet fasting period and semaglutide last taken 7 days prior revealed no food residue in stomach.	No aspiration event during EGD when food residue in stomach noted and procedure aborted
Giron-Arango et al. A A Pract. 2024 [51]	Case report	1 (74 yo M)	BMI 36	Type 2 diabetes, noted to not have history of GERD and gastroparesis	Semaglutide 1 mg subcutaneously weekly	Diabetes	14 h solids, 5 h fluid	Not held, last dose day prior to surgery	Not mentioned	Increased gastric volume assessed by gastric ultrasound consistent with full stomach, given metoclopramide, performed urological procedure under spinal without sedation after risk/benefit discussion with patient	None
Ishihara et al. Curues. 2022 [53]	Case report	1 (74 yo F)	Not mentioned	Type 2 diabetes (A1c 8.2); of note, also taking sitagliptin (DPP-4 inhibitor)	Liraglutide 0.6 mg (frequency not noted), started taking 4 days prior to symptoms onset	Not specifically noted, likely diabetes	Not mentioned	Not mentioned	Nausea, decreased appetite, abdominal distention, sore throat	NGT placed with 600 cc output, A gastroscopy was performed to investigate the cause of nausea, which showed a patent pylorus along with reflux esophagitis. An NGT was placed with 600 mL of output. EGD to workup nausea was remarkable for reflux esophagitis and no obstruction. CT abdomen revealed fluid accumulation from the stomach to the duodenum without bowel dilation or obstruction. Nausea resolved by third day after NGT placed and suctioned, metoclopramide treatment, and halting diabetic medications and oral nutrition.	Not mentioned
Gulak et al. Can J Anesth. 2023 [52]	Case report	1 patient (48 yo F)	BMI 28	Hypothyroidism, on levothyroxine. Noted to not be diabetic	Semaglutide 0.5 mg subcutaneous weekly, prescribed 5 months ago	Weight loss	20 h for solids, 8 h for clears	No withholding, last taken 2 days prior to surgery	No gastrointestinal side effects	OGT placed after regurgitation of excess of 200 mL of clear fluid following induction drained minimal gastric content.	The patient regurgitated a large volume, greater than 200 mL of clear fluid, which occurred after less than 30 s of gentle mask ventilation following standard sequence induction for a scheduled breast procedure. The patient was turned to the lateral decubitus position, suctioned, and then intubated. A flexible fiberoptic bronchoscopy revealed no evidence of aspiration. The surgery proceeded without complications, and the patient’s oxygenation and ventilation remained stable throughout. The patient was administered, metoclopramide and an OGT was placed before extubation, draining minimal gastric content. The patient was successfully extubated, transferred to postanesthesia care unit (PACU), and was discharged home the next day in stable condition.
Klein et al. Can J Anesth. 2023 [54]	Case report	1 (42 yo M)	BMI 37	Barrett’s esophagus, on PPI and histamine receptor antagonist; OSA, on CPAP; mixed anxiety and depressive disorder, on medications with anticholinergic effects (paroxetine); prior history of lung abscess likely secondary to aspiration in setting of heavy alcohol use, now sober for 4 years. Of note, patient did not have diabetes	Semaglutide 1.7 mg subcutaneous weekly, started taking two months ago with dosing progressively increased to current dosing regimen	Weight loss	>18 h	Not mentioned.	None mentioned	The patient underwent a repeat EGD for treatment of dysplastic mucosa under deep sedation where he was found to have copious quantities of liquid and solid in the stomach and experienced an aspiration event noted in the next column, which was different compared to his prior EGDs which were uncomplicated and his stomach was empty.	Following large quantities of liquid and solid in the stomach on EGD, the stomach was suctioned and the patient was intubated using a rapid sequence induction with succinylcholine. Bronchoscopy was performed, and food remains were removed from the trachea and bronchi. The patient was transferred to the ICU intubated and sedated, and subsequently extubated four hours later with no sequelae, remained asymptotic and discharged the next day.
Rai et al. Cureus. 2018 [55]	Case report	1 (52 yo M)	Not mentioned	Type 2 diabetes (A1c 7.0) Noted to have not been taking narcotics prior to and during hospitalization	Liraglutide 1.2 mg subcutaneously daily, duration not specified other than recently started	Diabetes	Not mentioned	Not mentioned	Nausea, abdominal distention, abdominal pain	NGT placed with 1 L fluid suctioned. EGD unremarkable except for mild irritation in the gastric body likely related to trauma from nasogastric tube. Resolution of symptoms with conservative management including discontinuation of liraglutide, gastric suctioning, anti-emetic and prokinetic therapy	Not mentioned
Weber et al. British Journal of Anaesthesia. 2023 [56]	Correspondence with editor—case report	1 (59 yo F)	Obesity and BMI not noted	No diabetes	Tirzepatide (dose and frequency not mentioned), no details on duration other than the drug was recently started	Weight loss	Noted to be appropriate	Not mentioned	Not mentioned	OGT placed after gastric aspirate in oropharynx triggered conversion from general anesthesia with an LMA to endotracheal tube (details in next column); patient had a large volume emesis that resulted in a total of 750 mL of undigested food and gastric contents suggesting delayed gastric emptying.	The patient underwent a hysteroscopy with polyp resection and experienced significant respiratory and gastrointestinal complications. Initially, a supraglottic airway was placed, but gastric aspirate was observed in the oropharynx shortly thereafter, necessitating tracheal intubation. Following intubation, the patient experienced a large volume emesis containing undigested food. An OGT was then inserted, removing an additional 500 mL of thick gastric aspirate. At the procedure’s conclusion, approximately 750 mL of undigested food and gastric contents were collected, indicating a significant aspiration event. Despite these complications, a subsequent chest radiograph revealed no signs of pneumonia or pneumonitis. The patient was admitted for overnight monitoring and was able to be weaned to room air quickly.

## Data Availability

No new data were created or analyzed in this study, as it is based entirely on previously published sources.

## Supplementary Materials

The following supporting information can be downloaded at: https://www.mdpi.com/article/10.3390/jcm13216336/s1, Supplementary Text S1: Summary of Patient Demographics and Clinical Characteristics related to age and sex demographics, BMI distribution, medication regimens, medication dosing regimens and durations, indication of GLP-1 RA, and fasting time prior to procedures; Supplementary Text S2: Detailed information for Case Series and Reports related to Gastrointestinal Symptoms and Risk for Retained Gastric Contents.
