# Supplementary material for: A Scoping Review of GLP-1 Receptor Agonists: Are They Associated with Increased Gastric Contents, Regurgitation, and Aspiration Events?
Source: J Clin Med. 2024 Oct 23;13(21):6336. doi: 10.3390/jcm13216336 (PMC11546377; doi:10.3390/jcm13216336)
Supplement: Supplementary file 1 [file jcm-13-06336-s001.zip › GLP-1 RA REVIEW - Supplementary Text S1.docx]

**Summary of Patient Demographics and Clinical Characteristics related to age and sex demographics, BMI distribution, medication regimens, medication dosing regimens and durations, indication of GLP-1 RA, and fasting time prior to procedures**

Age and Sex Demographic data

Prior studies have shown that age and sex may be associated with delayed gastric emptying and gastroparesis, particularly older patients and females^57-59^.

Of the 4 prospective studies, 2 of the studies by Quast et al. and Nakatani et al. had median ages of approximately 60 years old in both the GLP-1 receptor agonist user and non-user groups^46,47^; the remaining studies by Sen et al. and had a median age of 59 years old in the GLP-1 RA group and 53 years old in the control group^50^, and Sherwin et al had a median age of 41.5 years old in the semaglutide group and 31.5 years old in control group^51^. The majority of subjects in the prospective studies by Sherwin et al., Nakatani et al., and Quast et al.; were relatively more females in the GLP-1 RA group than the control group in the Quast et al.^47^, relatively less females in the GLP-1 RA group than the control group in the Sherwin et al. study^51^, and an equal number in both groups in the Nakatani et al. study^46^. The only prospective study to have a greater number of females was the study by Sen et al. which had a nearly equal number of females in both the GLP-1 RA and control groups^50^.

Of the six retrospective studies, the two studies by Stark et al. and Kobori et al. had a mean or median age of the patients being greater than 60 years old^45,53^; the study by Wu et al. had a median age of 64.1 years old in the GLP-1 RA group and 58.5 years old in the control group^56^; the studies by Bi et al. and Silveira et al. had mean and median ages of 55 and 50.8 years old, respectively^36,52^; and the study by Anazco et al did not report the age and sex distribution of the patients^34^. Bi et al. and Wu et al. were the only retrospective study in which a majority of the patients were females^36,56^; a majority of the patients were male in the study by Stark et al. and there was a nearly equal number of female and males in the study by Silveira et al^52,53^.

Of the five case series, the two case series by Raven et al and Kittner et al. had all 2 and 3 patients, respectively, of 60 years or greater^43,49^; one case series by Kalas et al. with both patients 45-60 years old^42^; one case series by Wilson et al. which had one patient 45-60 years old and one patient greater than 60 years old^55^; and one case series by Avraham et al. where one patient was 70 years old and the other 25 years old^35^. All the patients presented in the case series were females except the 2 studies by Kittner et al. (2 males and 1 female) and Avraham et al (1 female and 1 male)^35,43^.

Of the 9 case reports, 3 patients in the studies by Ishihara et al., Espinoza et al., and Giron-Arango et al. were 60 years old or greater^37,39,41^; 3 patients in the studies by Gulak et al., Webber et al., and Rai et al. were 45 to 60 years old^40,48,54^; and 3 patients in the studies by Klein et al., Almustanyir et al., and Fujino et al. were less than 45 years old^33,38,44^. The majority of the case report studies (6 of 9) were females with only the studies by Rai et al., Klein et al., and Giron-Arango being male^39,44,48^.

BMI Distribution

Of the 4 included prospective studies, 2 studies by Quast et al. and Sen et al. had a mean BMI of 30 to 35^47,50^; the other 2 studies by Sherwin et al. and Nakatani et al. had mean BMI less than 30^46,51^. Of the 6 retrospective studies, 2 studies by Stark et al. and Wu et al. had mean BMIs of 30 to 35^53,56^; 1 study by Silveira et al. had a median BMI less than 30^52^; and the other 3 studies by Kobori et al., Bi et al., and Anazco et al. did not report the BMI of the patients in their study^34,36,45^. Of the 5 case series, 4 consisted of patients with reported or presumed BMI 30 or greater (1 study by Wilson et al. with both patients with BMI >40^55^; 2 studies by Raven et al. and Avraham et al. with both patients with BMI of 30 to 35^35,49^; and 1 study by Kittner et al. with all 3 patients who were obese but did not have the BMI reported^43^), and 1 study by Kalas et al. where BMI was not reported^42^. Of the 9 case reports, 4 studies had a BMI of 30 or greater (2 studies by Fujino et al. and Espinoza et al. with BMI >40^37,38^; 2 studies by Klein et al. and Giron-Arango et al. with BMI 35-40^39,44^; and 1 study by Almustanyir et al. who had a BMI of 31.9^33^); 1 study by Gulak had a BMI <30^40^, and the remaining 3 studies by Ishihara et al., Rai et al., and Weber et al. did not report the BMI of their patients^41,48,54^.

Medication regimens

The reported medication regimens in the studied varied significantly and included semaglutide (both oral and subcutaneous formulations) liraglutide, dilaglutide, liraglutide, lixisenatide, exanataide, lixisenatide, and terzepatide. The reported dosing, frequency, and duration varied significantly amongst the studies and the specific details related to these medication regimen parameters were often times not reported. Nine of the fifteen studies that were not case reports had more than one formulations of GLP-1 receptor agonist taken by the patients in the study^34,42,45,47,49,50,53,55,56^. The medications used in order of most common to least common in the studies was: semaglutide (17 studies^34,35,37-40,42-45,49-53,55,56^), liraglutide (10 studies^33,34,41,45-49,53,56^), dilaglutide (7 studies^34,42,45,50,53,55,56^), exenatide (3 studies^34,45,53^), terzepatide (4 studies^34,50,54,56^), and lixisenatide (2 studies^34,45^). Of the prospective and retrospective studies, 1 study by Nakatani et al. had only liraglutide use^46^; 2 studies by Silveira et al. and Sherwin et al. had only semaglutide use^51,52^; 1 study by Quast et al. had lixisenatide and liraglutide use^47^; 1 study by Kobori et al. had dulaglutide, liraglutide, exenatide, and semaglutide use^45^; 1 study by Sen et al. had Semaglutide, dulaglutide, and tirzepatide use^50^; 1 study by Anazco et al. identified patients with lixisenatide, tirzepatide, exenatide, liraglutide, dulaglutide, and semaglutide use but did not specify which GLP-1 RAs were used^34^; 1 study by Wu et al. had semaglutide, liraglutide, dulaglutide, tirzepatide, and 2 combination of two different durgs^56^; 1 study by Stark et al. had dulaglutide, liraglutide, exenatide, and semaglutide use^53^; and 1 study by Bi et al. did not specify the GLP-1 receptor agonist used^36^.

Medication dosing regimens and durations

Of the 4 prospective studies, 1 study by Quast et al. involved manometry and pH studies prior to and after the use of either lixisenatide (10mcg daily for 1 week, followed by 20mcg daily for 10 weeks) or liraglutide (0.6mg daily for 1 week, followed by 1.2mg daily for week 2, followed by 1.8 mg daily) for a total duration of 10 weeks^47^; 1 study by Nakatani et al. involved capsule endoscopy studies prior to and after the use of liraglutide (0.3mg initially and titrated up to 0.3mg weekly to a final dose of 0.9mg weekly) for a total duration of 1 month^46^; 1 study by Sherwin et al. involved the use of gastric ultrasound in separate patients who were taking varying formulations (injectable and oral) and dosing (0.25 to 0.75mg) for varying durations (1-8 weeks), with the majority on less than 4 weeks of therapy at time of study enrollment^51^; and 1 study by Sen et al. evaluated retained gastric contents by preprocedural gastric ultrasound of patients taking semaglutide, dulaglutide, and tirzepatide (dosing and durations not specified)^50^. Of the 6 retrospective studies, all of which involved retrospective review of endoscopy studies, 1 study by Kobori et al. involved patients taking dulaglutide (0.75mg), liraglutide (<0.3 to 1.8mg), semaglutide (0.25-1.0mg), oral semaglutide (3-7mg), lixisenatide (<10 to 20mcg), exenatide (20mcg) (frequency and duration not noted)^45^; 1 study by Stark et al. involved endoscopies in patients taking liraglutide, exenatide, and semaglutide (dose, frequency, and duration of use not specified)^53^; 1 study by Silveira et al. involved the use semaglutide (dose, frequency and duration not reported^52^; 1 study by Wu et al. included patients taking semaglutide, liraglutide, dulaglutide, tirzepatide, and a combination of two drugs (dosing and durations not mentioned); and 1 study by Anazco et al. initially identified patients as having GLP-1 RA prescriptions (irrespective of endoscopy) for lixisenatide, tirzepatide, exenatide, liraglutide, dulaglutide, and semaglutide (dosing and duration not mentioned)^56^. Of the total of 18 patients in the 5 case series and 9 case reports, the majority (11 of 18 patients) were taking Semaglutide (1 patient on 7mg oral daily dosing with unspecified duration, 1 patient with 0.25mg weekly dosing for approximately 1 month, 1 patient with 0.5mg subcutaneous weekly dosing prescribed 5 months ago, 1 patient with 1mg dosing started a week prior to procedure, 2 patients with 1mg subcutaneous weekly dosing for unknown duration, 1 patient with 1.7mg subcutaneous weekly dosing with medication titration started 2 months ago, 4 patients with dosing and duration not mentioned); 4 of 14 patients taking liraglutide (1 patient with 0.6mg dosing started 4 days prior to NGT decompression, 1 patient with 1.2mg subcutaneous daily dosing with duration not specified other than recently started, and 2 patient with dosing and duration not mentioned); 2 of 14 patients taking dulaglutide (1 patient with 1.5mg subcutaneous weekly dosing with duration not specified and 1 patient with dosing and duration not mentioned), and 1 patient taking tirzepatide (dosing and duration not specified other than recently started). Of the 5 case series, Kittner et al. and Avraham et al. were the only studies where all the patients were using the same GLP-1 receptor agonist (all 5 patients used semaglutide)^35,43^; the other 3 case series involved the use of semaglutide and another GLP-1 receptor agonist which included dulaglutide or liraglutide.

Indication of GLP-1 receptor agonist

Of the 4 prospective studies, the indication for the GLP-1 receptor agonist in 2 studies by Quast et al. and Nakatani et al. was diabetes^46,47^; in the 1 study by Sherwin et al. was weightless except one patient in the GLP-1 receptor agonist group was taking for diabetes^51^; and 1 study by Sen et al. it was no specifically mentioned^50^. Of the 5 retrospective studies, the primary indication for GLP-1 receptor agonist in 1 study by Silveira et al. was weight loss in 87.8% patients followed by diabetes in 12.2% patients^52^; in 4 studies by Bi et al., Kobori et al., Stark et al., Anazco et al., and Wu et al. it was not specifically mentioned, however, it was noted in the Bi et al. study that patients with diabetics were specifically excluded from the GLP-1 receptor agonist analysis group^34,36,45,53^. Of the 5 case series, the indication for GLP-1 receptor agonist in the 1 study by Wilson et al. was diabetes for both patients^55^; and in the 3 studies by Raven et al., Kittner et al., Kalas et al., and Avraham et al. it was not specifically mentioned, however, it was noted in the Kittner et al. study that all patients were obese and had diabetes^35,42,43,49^. In the 9 case reports, the indication the indication for GLP-1 receptor agonists in 4 studies by Gulak et al., Klein et al., Webber et at., and Espinoza et al. was weight loss/obesity^37,40,44,54^; in the study by Almustanyir et al. was diabetes and obesity^33^; in the study by Rai et al. was diabetes^48^; and in the 1 study by Ishihara was not specifically noted, however, the patient was noted to have diabetes^41^.

Fasting time prior to procedures

Of the 4 prospective studies, the fasting times in 1 study by Naktani et al. was 11 hours^46^; 1 study by Sherwin et al. noted to be greater than 10 hours^51^; 1 study by Quast et al. was not specifically mentioned but noted fasting started at 8pm the day prior to the manometry and pH tests^47^; and 1 study by Sen et al. was not specifically mentioned aside from the patients followed standard preprocedural fasting guidelines^50^. Of the 6 retrospective studies, the fasting times in 1 study by Silveira et al. was a median fasting time (percentile 25-75%) for clear fluids of 9.3 (5.0-12.8) hours and 14.5 (12.2-28.7) hours for solids^52^; in 1 study by Wu et al. was a median time of 16 (IQR, 14-19) hours in the GLP-1 RA group and 16 (IQR, 14-19) hours in the control group^56^; and not specifically mentioned in the 4 studies by Kobori et al., Stark et al., Bi et al., and Anazco et al. with the Bi et al. study noting that they assumed adherence to standard pre-procedural fasting of 6-8 hours^34,36,45,53^. Of the 5 case series, the fasting times in 1 study by Raven et al. was 13 hours in one patient for the initial upper endoscopy where they observed a stomach full of solid food and 12 hours for repeat upper endoscopy after semaglutide was held for 3 weeks for one patient and 10 hours for the second patient^49^; 1 study by Kittner et al. was 11, 10, and 14 hours for three patients^43^; 1 study by Wilson et al. was 10 hours for solids and 4 hours for clear liquids in one patient and 16 hours for solids and 5 hours for clear liquids in a second patient^55^; 1 study by Avraham et al. where the fasting times for the two patients was 12 hours and > 8 hours^35^; and 1 study by Kalas et al. where the fasting times were not specifically mentioned for both patients^42^. For the 9 case reports, the fasting times was in 1 study by Fujino et al. 10 hours for solids for the first endoscopy and a 36 hour liquid fast for the repeat endoscopy^38^; 1 study by Gulak et al. reported 20 hours for solids and 8 hours for clears^40^; 1 study by Klein et al. reported greater than 18 hours^44^; 1 study by Giron-Arango et al. reported 14 hours for solids and 5 hours for fluid^39^; and 4 studies by Almustanyir et al., Rai et al., Ishihara et al., Weber et al., and Espinoza et al. did not specifically mentioned the fasting times, however, Weber et al. and Espinoza et al. noted the fasting times to be appropriate^33,37,41,48,54^.
