# Supplementary material for: A Scoping Review of GLP-1 Receptor Agonists: Are They Associated with Increased Gastric Contents, Regurgitation, and Aspiration Events?
Source: J Clin Med. 2024 Oct 23;13(21):6336. doi: 10.3390/jcm13216336 (PMC11546377; doi:10.3390/jcm13216336)
Supplement: Supplementary file 1 [file jcm-13-06336-s001.zip › GLP-1 RA REVIEW - Supplementary Text S2.docx]

**Detailed information for Case Series and Reports related to Gastrointestinal Symptoms and Risk for Retained Gastric Contents**

In the 4 case series that reported on the presence or absence of gastrointestinal symptoms, 1 study by Kalas et al. reported no remarkable findings on prior upper and lower endoscopies but had delayed gastric emptying on gastric emptying scintigraphy (GES) in the two reported patients both who had gastrointestinal symptoms^44^; 1 study by Kittner et al. revealed retained solids on gastric ultrasound in all three reported patients who all had gastrointestinal symptoms since starting GLP-1 RAs leading to case postponement^45^; 1 study by Wilson et al. reported perioperative regurgitation events in 2 patients who reported no gastrointestinal symptoms prior to procedure^47^; and 1 study by Avraham et al. that specifically mentioned gastrointestinal symptoms in 1 of 2 patients did not specifically report the gastric volume suctioned following nasogastric suctioning after a regurgitant episode during laryngoscopy^43^. In 5 case reports that reported on the presence or absence of gastrointestinal symptoms, 1 report by Fujino et al. reported retained gastric food contents in a patient without gastrointestinal symptoms^50^; 1 study by Gulak et al. reported in a patient without gastrointestinal symptoms that an orograstric tube placed after regurgitation of excess 200mL of clear fluid following induction drained minimal gastric content^52^; 1 study by Ishihara et al. reported in a patient with gastrointestinal symptoms had 600cc output after nasogastric tube placement and gastroscopy significant for reflux esophagitis^53^; 1 study by Rai et al. reported in a patient with gastrointestinal symptoms had 1L output after NGT placed and no pertinent findings on upper endoscopy^55^; and 1 study by Almustanvir et al. reported in a patient with gastroparesis symptoms with EGD with no significant findings^48^.
